# Novel spirooxindole-triazole derivatives: unveiling [3+2] cycloaddition reactivity through molecular electron density theory and investigating their potential cytotoxicity against HepG2 and MDA-MB-231 cell lines

**DOI:** 10.3389/fchem.2024.1460384

**Published:** 2024-09-30

**Authors:** Ihab Shawish, Samha Al Ayoubi, Ayman El-Faham, Ali Aldalbahi, Fardous F. El-Senduny, Farid A. Badria, Mar Ríos-Gutiérrez, Hassan H. Hammud, Sajda Ashraf, Zaheer Ul-Haq, Assem Barakat

**Affiliations:** ^1^ Department of Math and Sciences, College of Humanities and Sciences, Prince Sultan University, Riyadh, Saudi Arabia; ^2^ Chemistry Department, Faculty of Science, Alexandria University, Alexandria, Egypt; ^3^ Department of Chemistry, College of Science, King Saud University, Riyadh, Saudi Arabia; ^4^ Department of Pathology and Laboratory Medicine, Sylvester Comprehensive Cancer Center, Miller School of Medicine, Miami, FL, United States; ^5^ Department of Chemistry, Faculty of Science, Mansoura University, Mansoura, Egypt; ^6^ Department of Pharmacognosy, Faculty of Pharmacy, Mansoura University, Mansoura, Egypt; ^7^ Department of Organic Chemistry, University of Valencia, Valencia, Spain; ^8^ Department of Chemistry, College of Science, King Faisal University, Al-Ahsa, Saudi Arabia; ^9^ Dr. Panjwani Center for Molecular medicine and Drug Research, International Center for Chemical and Biological Sciences, University of Karachi, Karachi, Pakistan

**Keywords:** spirooxindole, 32CA reactions, anticancer, molecular electron density (MED), cytotoxcicity

## Abstract

A novel analogue of hybrid spirooxindoles was synthesized employing a systematic multistep synthetic approach. The synthetic protocol was designed to obtain a series of spirooxindole derivatives incorporating triazolyl-*s*-triazine framework via [3 + 2] cycloaddition (32CA) reaction of azomethine ylide (**AY)** with the corresponding chalcones **(6a-d)**. Unexpectedly, the reaction underwent an alternate route, leading to the cleavage of the s-triazine moiety and yielding a series of spirooxindole derivatives incorporating a triazole motif. A comprehensive investigation of the 32CA reaction mechanism was conducted using Molecular Electron Density Theory (MEDT). The viability of all compounds was evaluated through an MTT assay, and the IC_50_ values were determined using Prism Software. The antiproliferative efficacy of the synthesized chalcones and the corresponding spirooxindole derivatives was assessed against two cancer cell lines: MDA-MB-231 (triple-negative breast cancer) and HepG2 (human hepatoma). These findings were compared with Sorafenib, which was used as a positive control. The results revealed that chalcones **(6c** and **6d)** were the most active among the tested chalcones, with IC_50_ values of 7.2 ± 0.56 and 7.5 ± 0.281 µM for **(6c)** and of 11.1 ± 0.37 and 11.0 ± 0.282 µM for **(6d)**, against MDA-MB-231 and HepG2, respectively. Spirooxindoles **(9b, 9c, 9h,** and **9i)** exhibited the highest activity with IC_50_ values ranging from 16.8 ± 0.37 µM to 31.3 ± 0.86 µM against MDA-MB-231 and 13.5 ± 0.92 µM to 24.2 ± 0.21 µM against HepG2. In particular, spirooxindole derivatives incorporating 2,4-dichlorophenyl moiety were the most active, with an IC_50_ of 16.8 ± 0.37 µM for **(9h)** against MDA-MB-23 and 13.5 ± 0.92 µM for **(9i)** against HepG2. Interestingly, the IC_50_ of compound **(6c)** (7.2 µM) exhibited better activity than that of Sorafenib (positive control) (9.98 µM) against MDA-MB-231. Molecular docking, ADMET, and molecular dynamic simulations were conducted for the promising candidates (**6b, 6c,** and **9h)** to explore their binding affinity in the EGFR active site.

## Graphical Abstract

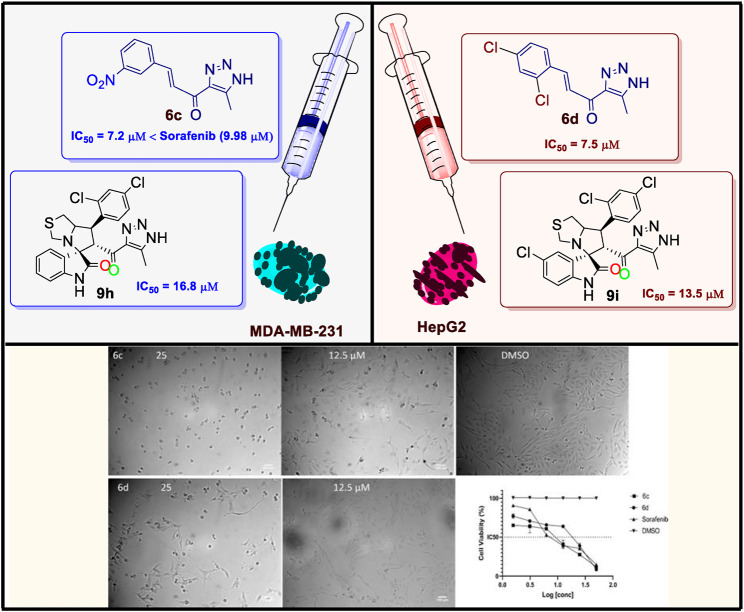

## 1 Introduction

**FIGURE 1:**
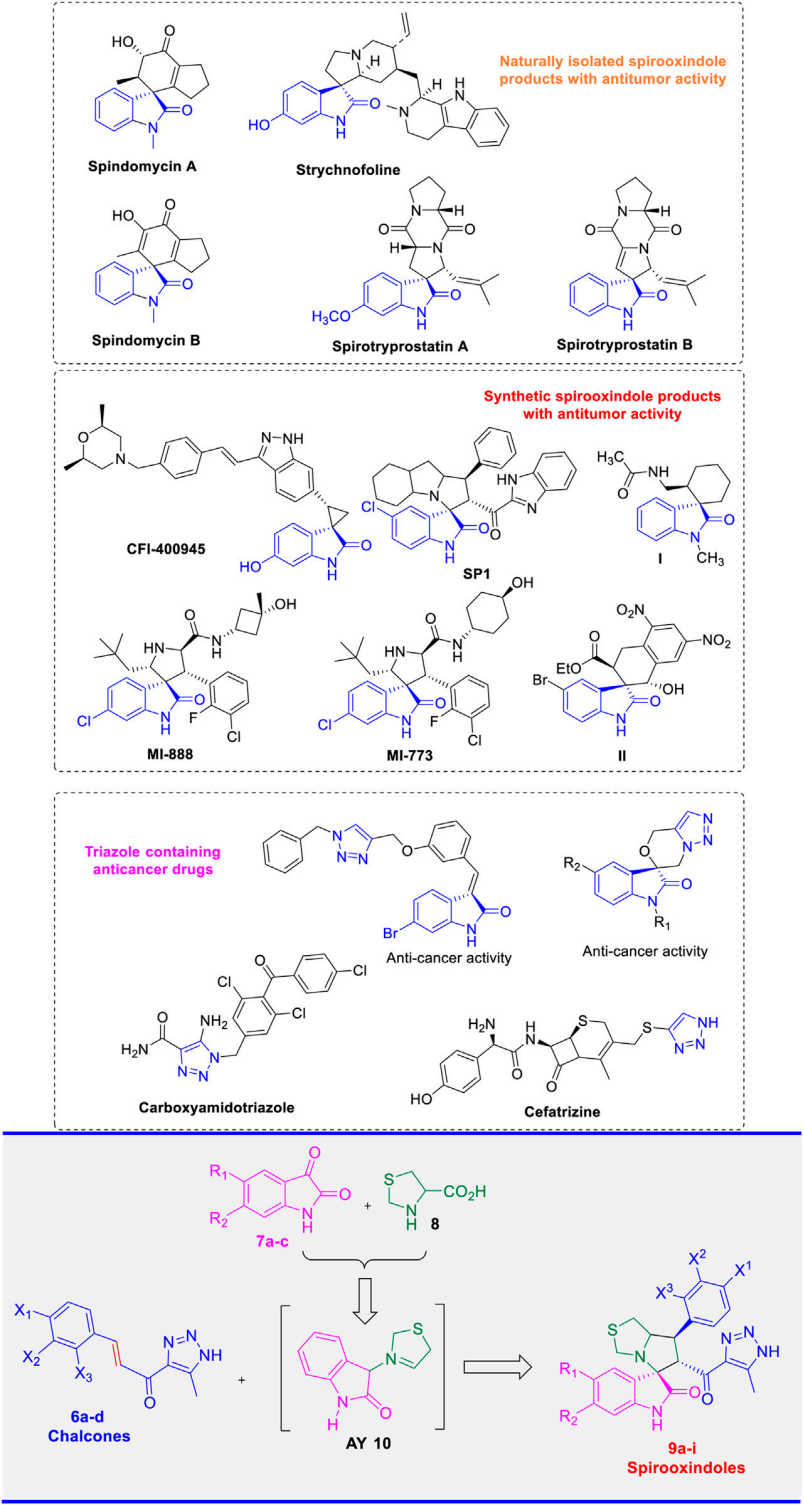
Naturally/synthetic spirooxindole and triazole lead molecules with antitumor activity and our designed derivatives **(9a-i)**.

The development of chemotherapeutic agents demonstrating high efficacy against cancer cells has been a focal point of research within the scientific community. Consequently, significant efforts have been made by chemists over the past decades to design innovative analogues with selective anticancer activity. (Modica-Napolitano and Weissig, 2015). In this regard, natural spirooxindole alkaloids were first isolated from different plant sources, such as Apocynaceae and Rubiacae, (Huang et al., 2019; Berger et al., 2022), which are considered among the most effective natural products with prominent activity against a wide spectrum of cancers. (Gouveia et al., 2019; Galliford and Scheidt, 2007; Panda et al., 2017). Additionally, these natural alkaloids have demonstrated significant activity in various pharmacological applications, including antimicrobial, (Yang et al., 2018), antifungals, (Fausto et al., 2019), antimycobacterial, (Senthilkumar et al., 2009), cholinesterase inhibitors, (Ming et al., 2023), and anti-inflammatory activity. (Ming et al., 2023). The diverse biological activity of these compounds is attributed to their unique structural framework, which features a spiro ring fusion at the position-3 of the oxindole (Helal et al., 2024). The multifunctional oxindole moiety can act as both a hydrogen bond donor or acceptor, thereby enhancing the interaction with different biological targets. Additionally, the multi-optional combination with a variety of bioactive cycloalkyl or heterocyclic moieties results in a significant enhancement of their activity in a wide of applications. (Zhou et al., 2020). Some representative examples of spirooxindole frameworks, isolated from natural sources and exhibiting potent activity toward different types of cancer cells are shown in (Figure 1). Strychnofoline, a naturally occurring product with a spirooxindole scaffold, exhibited remarkable activity against melanoma and Ehrlich tumor cells. (Yu et al., 2018; Yuenyongsawad et al., 2013). Two spirooxindoles, spindomycins A and B, were isolated from the rhizosphere strain *Streptomyces* sp. xzqh-9 and reported as potential inhibitors of the tyrosine kinase BCR-ABL. (Guo et al., 2014). The natural product Spirobrassinin is an oxindole alkaloid that was isolated from *Daikon Raphanus sativus L*. var. and showed potent antitumor activity. (Budovská et al., 2020). Spirotryprostatins A and B are other representative examples of natural bioactive products incorporating a spirooxindole framework with prominent inhibitory activity against mouse breast cancer tsFT210. (Ding et al., 2005; Al-Rashood et al., 2020).

Owing to their diverse pharmacological applications, particularly as anticancer agents, natural spirooxindole alkaloids have inspired chemists to design and synthesize a vast array of synthetic spirooxindole analogues that mimic their structural frameworks. In this regard, compounds **I** and **II** (Figure 1 **)** are examples of novel spirooxindole-cyclohexane derivatives that have shown prominent activity both *in vitro* and *in vivo* against liver cancer (HepG2) and glioblastoma cell lines, respectively. (Morales-Ríos et al., 2020; Wang et al., 2020). **CFI-400945** is another lead spirooxindole derivative that demonstrates potent activity against PLK4 with a *K*_i_ value of 0.26 nM. Further advanced biological studies have identified this compound as a promising inhibitor of solid tumors, leading to its inclusion in phase I clinical trials (NCT01954316) as an anti-breast cancer agent. (Jacqueline et al., 2014). Recent *in vitro* and *in vivo* studies have indicated that the novel spirooxindole derivative which incorporates a benzimidazole moiety **SP1** exhibits potent anticancer activity, making it a promising candidate as a potential drug for the treatment of breast adenocarcinoma. (Barakat et al., 2022). Moreover, **MI-888** and **MI-773** are among the most important pyrrolidine-fused spirooxindole derivatives that have exhibited significant and selective inhibitory activity against MDM2-p53 interaction. (Zhao et al., 2013; de Jonge et al., 2017).

The construction of hybrid derivatives, that combine diverse scaffolds with potent anticancer activity, is a well-established protocol in medicinal chemistry. This approach holds a great potential for providing valuable therapeutic interventions for cancer treatment. Additionally, 1,2,3-triazole containing compounds have emerged as highly active heterocyclic pharmacophores, exhibiting remarkable activity against various types of cancer cells. (Liang et al., 2012; Sachdeva et al., 2022; Al Sheikh Ali et al., 2021; Ríos-Malváez et al., 2021). This pronounced bioactivity of this privileged structure can be attributed to its ability to interact with various enzymes, proteins, and receptors via several non-covalent bonds such as hydrogen bonds, dipole-dipole interactions, and Van der Waals forces. (Bozorov et al., 2019). Carboxyamido-triazole (CAI) and Cefatrizine (Figure 1) are representative examples of compounds containing a 1,2,3-triazole scaffold with anticancer potential. (Al Sheikh Ali et al., 2021; Xu et al., 2019). Indeed, spirooxindole (Senwar et al., 2015) and isatin (Nagarsenkar et al., 2016) scaffolds having triazole moiety have been found to be effective against cancer cells (Figure 1). (Bozorov et al., 2019) Spirooxindoles and triazoles have demonstrated a wide range of bioactivity against several types of cancer cells, including breast and HepG2 cancer cells. Numerous articles and reviews have addressed these findings. Recently, several spirooxindole analogues have been developed and evaluated, showing potential as biomarker apoptosis inducers and as inhibitors targeting EGFR and CDK2 enzymes. (Nafie et al., 2024; Barakat et al., 2023).

Building on the prior studies conducted by Barakat and colleagues, which focused on developing novel hybrids of spirooxindoles as potential anticancer agents, (Islam et al., 2023; Al-Jassas et al., 2023), the present project was designed to synthesize a novel analogue based on spirooxindole scaffold incorporating a 1,2,3-triazole motif. The synthetic methodology involved a 32CA reaction of azomethine ylide intermediate **(AY)** with the corresponding chalcones **(6a-d)** to yield the target spirooxindole hybrids **(9a-i)** as shown in Figure 1.

An innovative theory in organic chemistry that builds on Density Functional Theory (DFT) is Molecular Electron Density Theory (MEDT). (Domingo, 2016). MEDT emphasizes changes in electron density to explain chemical reactivity, whereas DFT concentrates on ground-state electron density to identify molecular characteristics. (Domingo and Ríos-Gutiérrez, 2022; Chalkha et al., 2022; Daoui et al., 2023; Daoui et al., 2022). MEDT offers a contemporary approach for understanding and explaining experimental results by examining changes in electron density and their associated energy throughout the reaction path. This enables a deeper understanding of molecular mechanisms and reactivity. In this study, MEDT was employed to theoretically examine the reaction reactivity of the azomethine **(AY)** and the chalcone **(6m)** to produce the corresponding spirooxindole derivative **(9m)**. This investigation helps in understanding the properties of 32CA reactions. (Salah et al., 2021). In addition, the potential cytotoxicity of the target spirooxindole derivatives was evaluated against HepG2 (Liver) and MDA-MB-231 (Breast) cancer cell lines. This study also included molecular docking, ADMET analysis, and molecular dynamics simulations for the most active compounds.

## 2 Materials and methods

### 2.1 Chemistry

“Unless specified otherwise, reagents were obtained from commercial suppliers such as Sigma-Aldrich (Chemie GmbH, Taufkirchen, Germany) and used as received without further purification. Thin-layer chromatography (TLC) was conducted on Merck Silica Gel 60 F254 plates (20 cm × 20 cm) and visualized using a UV lamp at 254 nm. A standard rotary evaporator was used for vacuumed removal of the solvents. ^1^H- and ^13^C- Nuclear Magnetic Resonance spectra were recorded in CDCl_3_ and DMSO-*d*_*6*_ on a JEOL spectrometer (JEOL, Tokyo, Japan) (400 or 500 MHz) and referenced to residual solvent signals (*δ* 2.50 and 7.26 *δ*, respectively). ^1^H-NMR data were reported as chemical shifts (*δ* ppm) with signal multiplicity indicated as follows: s = singlet, d = doublet, t = triplet, q = quartet, m = multiplet, dd = doublet of doublets. Coupling constants (*J*) were reported in Hertz (Hz) and rounded to the nearest 0.1 Hz. Chemical shifts for ^13^C-NMR were also reported as (δ ppm). Infrared spectra were obtained using a Thermo Scientific Nicolet iS10 FT-IR spectrometer (Thermo Fisher Scientific, Waltham, MA, United States).”

#### 2.1.1 General procedure for the synthesis of 1,2,3-triazolyl-s-triazene derivatives, 4a-b

2-Azido-4,6-dichloro-*s*-triazene **(2)** was prepared by adding cyanuric chloride solution **(1)** (1 equivalent) in MeCN to a solution of sodium azide, NaN_3_ (1 equivalent) in water, while stirring at 0°C in an ice bath. The reaction was complete within 30 min, as indicated by TLC analysis. The work-up procedure involved removing the MeCN solvent under reduced pressure followed by extraction of the aqueous layer with cold DCM. The organic layer was dried over anhydrous MgSO₄ and then evaporated under reduced pressure. The resulting crude azido derivative was subsequently purified via column chromatography (ethyl acetate/*n*-hexane, 1:4) to afford the target 2-azido-4,6-dichloro-1,3,5-triazene **(2)** as a white solid, achieving a 75% yield. Subsequently, the intermediate 2-Azido-4-chloro-6-methoxy-1,3,5-triazine was prepared by dissolving 2-azido-4,6-dichloro-1,3,5-triazene **(2)** (1 equivalent) in excess methanol. Sodium bicarbonate (1 equivalent) was then added with stirring at room temperature for 30 min, until the evolution of CO₂ ceased. Excess water was added after the reaction was complete, and the product was then extracted using DCM. Subsequently, the extract was dried over anhydrous MgSO_4_, and the organic layer was evaporated under vacuum, yielding the target compound as a white solid with no further purification and achieving a 98% yield.

2-Azido-4,6-dimethoxy-1,3,5-triazine **(3a)** was prepared by using two equivalents of sodium bicarbonate. 2-Azido-4-methoxy-6-(piperidin-1-yl)-1,3,5-triazine **(3b)** was synthesized by adding piperidine (1 equivalent) dropwise to a solution of 2-azido-4-chloro-6-methoxy-1,3,5-triazine in acetone, with stirring at room temperature. Subsequently, a sodium bicarbonate solution (1.1 equivalent) in water was added, and the reaction mixture was stirred at room temperature for 2 h, as indicated by TLC analysis. Excess water was then added, causing the product to precipitate as a white solid, which was filtered, dried, and collected without further purification.

1,2,3-Triazolyl-s-triazene derivatives **(4a-b)** were synthesized by adding the corresponding azido-triazine derivative **(3a** or **3b)** (1 equivalent) to a stirred solution of acetylacetone (1.2 equivalent) and TEA (1.2 equivalent) in DMF. TLC (CHCl₃/MeOH, 9:1) was used to monitor the reaction, which was complete after 2–3 h. Excess water was then added to precipitate the product(s) **(4a-b)** as an off-white solid, which was filtered, washed with n-hexane, and dried. The yield of this reaction was 85%–90%, and the products were collected in pure form and used directly in the next step.

1,2,3-Triazolyl-s-triazene derivative, **4a**.


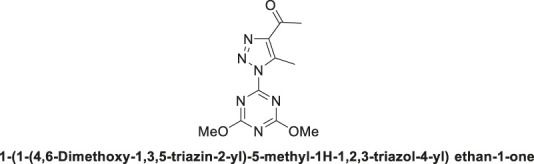


Pale-yellow solid in 90% chemical yield; m.p. 201°C–203°C; ^1^H NMR (500 MHz, CDCl_3_) *δ* 4.13 (s, 6H, 2CH_3_O), 2.98 (s, 3H, CH_3_), 2.73 (s, 3H, CH_3_C = O); ^13^C NMR (126 MHz, CDCl_3_) *δ* 194.4 (C=O), 173.5, 164.7 (C-Triazine), 144.1, 139.9 (2C-Triazole), 56.3 (2CH_3_O), 28.6 (CH_3_C = O), 12.0 (CH_3_-triazole); Anal. Calc. for C_10_H_12_N_6_O_3_ (264.25); C, 45.45; H, 4.58; N, 31.80. Found C, 45.33; H, 4.49; N, 31.75.

1,2,3-Triazolyl-s-triazene derivative, **4b**.


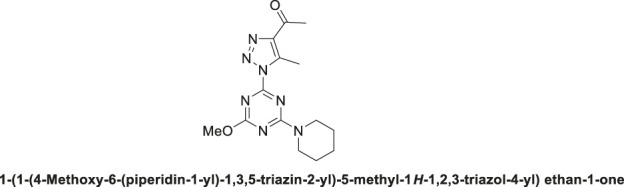


Off-white solid with 89% chemical yield: m.p. 195°C–197°C; ^1^H NMR (500 MHz, CDCl_3_) *δ* 4.00 (s, 3H, OCH_3_), 3.84 (t, *J* = 5.6 Hz, 4H, 2-CH_2_N), 2.89 (s, 3H, CH_3_), 2.70 (s, 3H, CH_3_C = O), 1.73–1.65 (m, 2H, CH_2_-Pip.), 1.65–1.57 (m, 4H, 2CH_2_-Pip.); ^13^C NMR (126 MHz, CDCl_3_) *δ* 194.6 (C=O), 171.9, 165.5, 163.4 (3C-triazine), 143.9, 139.3 (2C-Triazole), 55.2 (CH_3_O), 45.4, 45.1 (2CH_2_N), 28.4, 25.8, 25.7, 24.5, 11.8; Anal. Calc. for C_14_H_19_N_7_O_2_ (317.35); C, 52.99; H, 6.03; N, 30.90. Found C, 53.12; H, 5.98; N, 30.99.

#### 2.1.2 General procedure for the synthesis of 1,2,3-triazolyl-chalcones, 6a-d

Disubstituted-1,3,5-triazin-2-yl)-5-methyl-1*H*-1,2,3-triazol-4-yl) ethan-1-one derivatives **(4a-b)** (1 equivalent) were dissolved in ethanol with gentle warming until fully dissolved. A solution of 7% (w/v) KOH was then gradually added with stirring at rt for 5 min. The mixture was cooled to 0°C in an ice bath, and the benzaldehyde derivative **(5a-d)** was added portion-wise with stirring for 1–2 h. The reaction progress was monitored by TLC analysis (ethyl acetate/n-hexane 4:6), which showed the formation of the desired chalcones along with traces of side products. Subsequently, the organic solvent was evaporated, and the product was extracted using ethyl acetate (3 mL × 15 mL). The aqueous layer was then acidified with 10% (v/v) HCl, and DCM (3 mL × 15 mL) was used to extract the residual chalcone. The organic solvents were collected, dried over magnesium sulfate, and evaporated under vacuum. The crude product was purified by column chromatography (ethyl acetate-petroleum ether 3:7) to yield the target chalcones (6a-d) as a pale-yellow solid with a good yield (60%–75%).

1,2,3-Triazolyl-chalcone, **6a**.


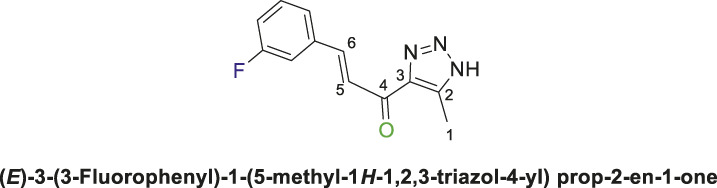


To a solution of 1-(1-(4,6-dimethoxy-1,3,5-triazin-2-yl)-5-methyl-1*H*-1,2,3-triazol-4-yl) ethan-1-one **(4a)** (528 mg, 2.0 mmol) in 30 mL ethanol, 7 mL of 7% (w/v) KOH solution was added dropwise. After stirring the reaction mixture for 10 min at rt, it was transferred to an ice bath and cooled to 0°C. Subsequently, 3-fluorobenzaldehyde **(5a)** (248 mg, 2.0 mmol) was added, and the mixture was stirred for 1 h. Upon completion of the reaction, as confirmed by TLC analysis, the product was extracted and purified following the general procedure of compounds **(6a-d)**, yielding the chalcone **(6a)** as a pale-yellow solid (325 mg, 1.40 mmol, 70%); m.p. 210°C–213°C; ^1^H NMR (500 MHz, DMSO-_*d6*_) *δ* 15.5 (s, 1H, NH), 7.89 (s, 1H), 7.71 (d, *J* = 10.9 Hz, 1H), 7.56 (d, *J* = 12.6 Hz, 2H), 7.40 (d, *J* = 28.1 Hz, 1H), 7.20 (d, *J* = 17.8 Hz, 1H) (4 Aromatic Protons and H-C5, H-C-6), 2.39 (s, 3H, 3H-C1); ^13^C NMR (101 MHz, DMSO-_*d6*_) *δ* 183.8(C=O), 163.8, 162.0, 129.9, 126.3, 124.8, 123.2, 117.7, 116.7, 115.4, 114.7 (6 aromatic carbons + C2,3,5,6), 27.95 (C1); Anal. Calc. for C_12_H_10_FN_3_O (231.23); C, 62.33; H, 4.36; N, 18.17. Found C, 62.23; H, 4.41; N, 18.11.

Chalcone **(6a)** was also synthesized by following the previously described procedure, using *(E)*-3-(3-fluorophenyl)-1-(1-(4-methoxy-6-(piperidin-1-yl)-1,3,5-triazin-2-yl)-5-methyl-1*H*-1,2,3-triazol-4-yl) prop-2-en-1-one **(4b)** instead of **(4a).** The obtained chalcone was collected as a pale-yellow solid in 66.7% yield; m.p.189°C–190°C; ^1^H NMR (400 MHz, DMSO-_*d6*_) *δ* 15.40 (m, 1H, NH), 7.97 (d, *J* = 16.2 Hz, 1H), 7.75 (d, *J* = 15.9 Hz, 1H), 7.60 (d, *J* = 8.1 Hz, 2H), 7.53–7.36 (m, 1H), 7.24 (t, *J* = 8.4 Hz, 1H) (4 Aromatic Protons and H-C5, H-C-6), 2.30 (s, 3H, 3H-C1). ^13^C NMR (126 MHz, DMSO-_*d6*_) *δ* 183.87(C=O), 164.0, 162.0, 131.3, 127.0, 125.20, 124.9, 117.6, 116.8, 115.4, 115.2 (6 aromatic carbons + C2,3,5,6), 28.0 (C1); Anal. Calc. for C_12_H_10_FN_3_O (231.23); C, 62.33; H, 4.36; N, 18.17. Found C, 62.40; H, 4.38; N, 18.26.

1,2,3-Triazolyl chalcone, **6b**.


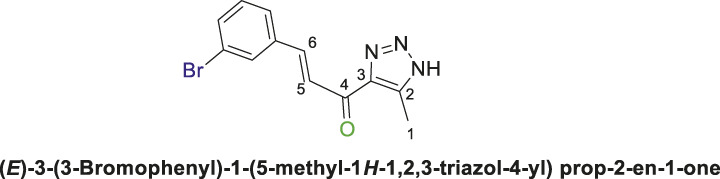


1,2,3-Triazolyl-*s*-triazene derivatives (**4a** or **4b)** and 3-bromobenzaldehyde **(5b)** were used as starting materials to obtain chalcone **(6b)** as a pale-yellow precipitate with 65% chemical yield, m.p. 193°C–195°C; ^1^H NMR (500 MHz, DMSO-_*d6*_) *δ* 15.38 (s, 1H, NH), 8.01 (q, J = 1.8 Hz, 1H), 7.94–7.92 (m, 1H), 7.82–7.80 (m, 1H), 7.76–7.70 (m, 1H), 7.69–7.56 (m, 1H), 7.39–7.36 (m, 1H) (4 Aromatic Protons and H-C5, H-C-6), 2.40 (s, 3H, 3H-C1), 13C NMR (126 MHz, DMSO-d6) δ 183.9 (C=O), 141.5, 137.5, 136.1, 133.7, 131.7, 131.6, 131.4, 127.9, 125.0, 122.9(6 Aromatic Carbons + C2,3,5,6), 28.1 (C1); Anal. Calc. for C12H10BrN3O (292.14); C, 49.34; H, 3.45; N, 14.38. Found C, 49.21; H, 3.49; N, 14.50.

1,2,3-Triazolyl chalcone, **6c**.


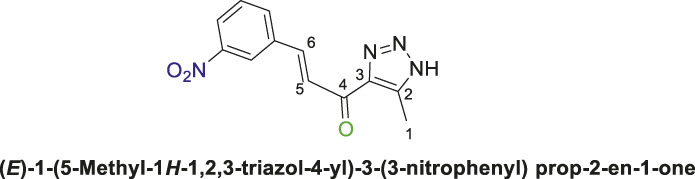


Compounds **(4a** or **4b)** and 3-nitrobenzaldehyde **(5c)** were reacted according to the previous procedure to obtain the chalcone **(6c)** as pale-yellow solid in 68% yield; m.p. 207°C–210°C; ^1^H NMR (400 MHz, DMSO-_*d6*_) *δ* 12.51 (s, 1H, NH), 8.57 (s, 1H), 8.26 (d, *J* = 8.7 Hz, 2H), 8.14–7.97 (m, 1H), 7.97–7.79 (m, 1H), 7.61 (dt, *J* = 14.8, 8.4 Hz, 1H) (4 Aromatic Protons and H-C5, H-C-6), 2.43 (s, 3H, 3H-C1); ^13^C NMR (101 MHz, DMSO-_*d6*_) *δ* 184.2 (C=O), 149.7, 148.7 (d, *J* = 43.3 Hz), 144.0, 140.9, 136.0, 133.8, 131.1, 126.1, 125.6, 124.1 (6 Aromatic C + C2,3,5,6), 27.0 (C1); Anal. Calc. for C_12_H_10_N_4_O_3_ (258.24); C, 55.81; H, 3.90; N, 21.70. Found C, 55.94; H, 3.79; N, 21.81.

12,3-Triazolyl chalcone, **6d**


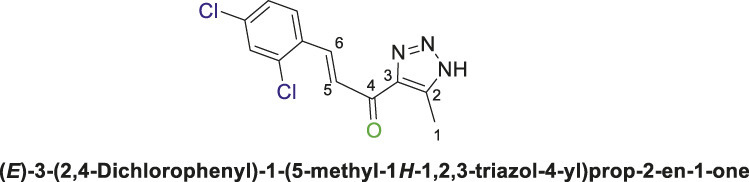


2,4-Dichlorobenzaldehyd **(5d)** and the derivative **(4a** or **4b)** were reacted to afford the target chalcone **(6d)** as an off-white solid in 73% yield; m.p. 220°C–22°C; ^1^H NMR (400 MHz, DMSO-_*d6*_) *δ* 8.01 (d, *J* = 16.0 Hz, 1H), 7.95–7.81 (m, 2H), 7.52 (dt, *J* = 14.2, 6.7 Hz, 1H), 7.38 (d, *J* = 16.6 Hz, 1H) (3 Aromatic Protons and H-C5, H-C-6), 2.39 (s, 3H, 3H-C1), ^13^C NMR (101 MHz, DMSO-_*d6*_) *δ* 183.6 (C=O), 142.5, 139.2, 136.6, 136.1, 135.6, 131.7, 130.0, 128.9, 128.6, 126.8 (6 Aromatic C + C2,3,5,6), 28.0 (C1); Anal. Calc. for C_12_H_9_Cl_2_N_3_O (282.12); C, 51.09; H, 3.22; N, 14.89. Found C, 51.00; H, 3.31; N, 14.93.

#### 2.1.3 General procedure for the synthesis of 1,2,3-triazolyl-spirooxindole derivatives, 9a-i

(s)-Thiazolidine-4-carboxylic acid **(8)** (1 equivalent) was added to a solution of isatin **(7a)** or monochloroisatin **(7b** or **7c)** (1 equivalent) in methanol, and the mixture was stirred at rt for 15 min. Thereafter, chalcone **(6a-d)** was added and the reaction was refluxed for 12 h in an oil bath. Upon completion of the reaction as indicated by TLC analysis (ethyl acetate/*n*-hexane 6:4), the solvent was evaporated under reduced pressure. The crude product was purified by column chromatography (ethyl acetate-petroleum ether 4:6) to afford the spirooxindole derivatives **(9a-i)** as an off-white solid with a yield of (75%–90%). In some cases, the product was initially oily after solvent evaporation and required dissolution in diethyl ether, followed by the addition of a small amount of *n*-hexane to yield the target spirooxindole derivative as an off-white precipitate.

Spirooxindole derivative, **9a**.


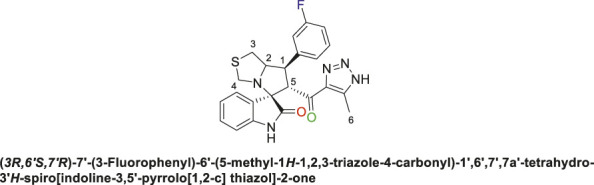


Off-white solid in 94% yield; m.p. 201°C–204°C; ^1^H NMR (400 MHz, DMSO-_*d6*_) *δ* 10.33 (s, 1H, NHC = O), 7.42–7.23 (m, 3H), 7.15 (d, *J* = 7.7 Hz, 1H), 7.03 (q, *J* = 8.8, 8.1 Hz, 2H), 6.81 (t, *J* = 7.5 Hz, 1H), 6.51 (d, *J* = 7.7 Hz, 1H), (**8 aromatic protons**), 4.83 (d, *J* = 10.9 Hz, 1H, H-C5), 4.19 (dt, *J* = 10.2, 5.3 Hz, 1H, H-C2), 3.96–3.87 (m, 1H, H-C1), 3.65 (d, *J* = 8.8 Hz, 1H, H-C4), 3.39 (d, *J* = 8.7 Hz, 1H, H-C4), 2.99 (dd, *J* = 10.7, 6.1 Hz, 1H, H-C3), 2.89 (dd, *J* = 10.7, 4.8 Hz, 1H, H-C3), 1.96 (s, 3H, 3H-C6); ^13^C NMR (101 MHz, DMSO-_*d6*_) *δ* 191.3 (C=O), 178.9 (-NHC = O), 164.1 (C-F), 161.7, 143.5, 143.0, 142.2, 131.1, 130.0, 127.4, 124.5, 121.2, 115.3, 115.0, 114.3, 109.8, 74.3 (shared spiro C), 72.1 (C2), 65.1 (C4), 51.7 (C5), 48.7 (C3), 35.2 (C1), 9.6 (C6); Anal. Calc. for C_23_H_20_FN_5_O_2_S (449.50); C, 61.46; H, 4.48; N, 15.58. Found C, 61.39; H, 4.51; N, 15.39.

Spirooxindole derivative, **9b**.


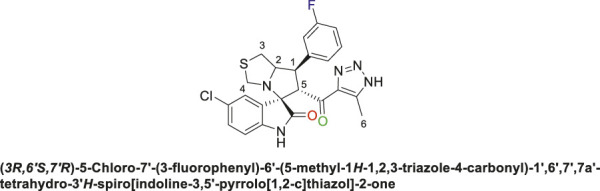


Off-white solid in 70% yield; m.p. 222°C–225°C; ^1^H NMR (400 MHz, DMSO-_*d6*_) *δ* 15.30 (s, 1H, NH), 10.54 (s, 1H, NHC = O), 7.41 (q, *J* = 7.3 Hz, 1H), 7.33 (d, *J* = 8.2 Hz, 2H), 7.15 (d, *J* = 7.7 Hz, 2H), 7.10 (dt, *J* = 9.4, 4.6 Hz, 1H), 6.55 (d, *J* = 8.3 Hz, 1H) (7 Aromatic protons), 4.89 (s, 1H, H-C5), 4.16 (dt, *J* = 10.2, 5.6 Hz, 1H, H-C2), 3.92 (t, *J* = 9.8 Hz, 1H, H-C1), 3.65 (d, *J* = 9.2 Hz, 1H, H-C4), 3.32 (d, *J* = 8.8 Hz, 1H, H-C4), 3.01 (dd, *J* = 10.7, 6.1 Hz, 1H, H-C3), 2.94 (dd, *J* = 10.9, 4.7 Hz, 1H, H-C3), 2.04 (s, 3H, 3H-C6), ^13^C NMR (126 MHz, DMSO-_*d6*_) *δ* 191.3 (C=O), 179.2 (-NHC = O), 163.8 (C-F), 161.8, 142.8, 141.9, 141.4, 131.4, 130.2, 127.5, 125.9, 125.6, 124.7, 115.4, 114.6, 111.5 (14 aromatic carbons), 74.4 (shared spiro C), 73.2 (C2), 64.5 (C4), 52.9 (C5), 49.3 (C3), 35.7 (C1), 9.1 (C6); Anal. Calc. for C_23_H_19_ClFN_5_O_2_S (483.95); C, 57.08; H, 3.96; N, 14.47. Found C, 57.18; H, 4.02; N, 14.51.

Spirooxindole derivative, **9c**.


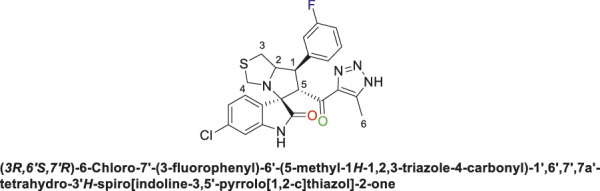


Pale-yellow solid with 86% yield; m.p. 192°C–195°C; ^1^H NMR (400 MHz, DMSO-_*d6*_) *δ* 10.51 (s, 1H, NHC = O), 7.37 (s, 1H), 7.26 (s, 2H), 7.06 (s, 2H), 6.89 (s, 1H), 6.53 (s, 1H) (7 Aromatic protons), 4.84 (d, *J* = 10.7 Hz, 1H, H-C5), 4.12 (s, 1H, H-C2), 3.88 (s, 1H, H-C1), 3.61 (d, *J* = 8.6 Hz, 1H, H-C4), 3.36 (s, 1H, H-C4), 2.98 (d, *J* = 10.9 Hz, 1H, H-C3), 2.88 (s, 1H, H-C3), 2.01 (s, 3H, 3H-C6), ^13^C NMR (126 MHz, DMSO-_*d6*_) *δ* 191.3 (C=O), 178.7 (-NHC = O), 163.8 (C-F), 161.8, 144.6, 141.9, 137.9, 134.3, 131.3, 128.8, 124.5, 123.4, 121.0, 115.5, 114.45, 109.7 (14 aromatic C), 74.3 (Shared C), 71.8 (C2), 65.0 (C4), 51.7 (C5), 49.0 (C3), 35.2 (C1), 8.7 (C6); Anal. Calc. for C_23_H_19_ClFN_5_O_2_S (483.95); C, 57.08; H, 3.96; N, 14.47. Found C, 57.03; H, 3.79; N, 14.49.

Spirooxindole derivative, **9d**


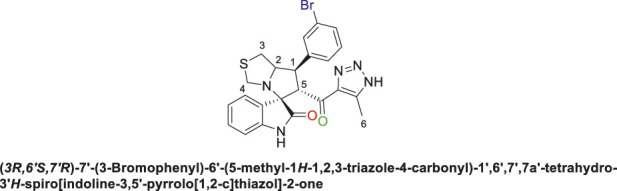


Off-white solid in 73% yield; m.p. 186°C–188°C; ^1^H NMR (400 MHz, DMSO-_*d6*_) *δ* 10.31 (s, 1H, NHC = O), 7.71–7,46 (s, 3H), 7.44–7.40 (d, J = 17.6 Hz, 2H), 7.21–6.78 (s, 2H), 6.43 (s, 1H) (8 aromatic protons), 4.81 (m, 1H, H-C5), 4.12 (s, 1H, H-C2), 3.91 (s, 1H, H-C1), 3.60 (s, 1H, H-C4), 3.51 (t, J = 6.9 Hz, 1H, H-C4), 2.98–2.87 (d, J = 22.0 Hz, 2H, 2H-C3), 1.95–1.84 (s, 3H, 3H-C6), ^13^C NMR (126 MHz, DMSO-_*d6*_) *δ* 191.4 (C=O), 178.8 (-NHC = O), 148.6, 143.1, 142.3, 135.6, 131.0, 130.2, 128.0, 122.8, 121.3, 116.7, 109.9 (aromatic C), 74.31 (Shared C), 72.0 (C2), 65.4 (C4), 52.3 (C5), 48.4 (C3), 34.1 (C1), 15.4 (C6); Anal. Calc. for C_23_H_20_BrN_5_O_2_S (510.41); C, 54.12; H, 3.95; N, 13.72. Found C, 54.08; H, 3.73; N, 13.87.

Spirooxindole derivative, **9e**.


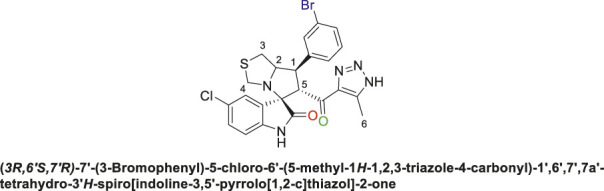


Pale-yellow solid in 70% yield; m.p. 206°C–208°C; ^1^H NMR (400 MHz, DMSO-_*d6*_) *δ* 15.26 (s, 1H, NH), 10.53 (s, 1H, NHC = O), 7.69 (s, 1H), 7.52 (s, 2H), 7.46 (s, 1H), 7.34–7.14 (s, 2H), 6.56 (s, 1H) (7 aromatic protons), 4.84 (s, 1H, H-C5), 4.17 (s, 1H, H-C2), 3.92 (s, 1H, H-C1), 3.64 (s, 1H, H-C4), 3.55 (s, 1H, H-C4), 2.97 (d, J = 22.0 Hz, 2H, 2H-C3), 2.06–1.93 (m, 3H, 3H-C6),^13^C NMR (101 MHz, DMSO-_*d6*_) *δ* 191.1 (C=O), 178.3 (-NHC = O), 148.6, 142.8, 142.1, 137.9, 135.5, 130.9, 130.0, 127.1, 126.5, 125.3, 123.4, 122.8, 111.2 (Aromatic C), 74.4 (Shared C), 72.0 (C2), 65.3 (C4), 51.4 (C5), 48.3 (C3), 34.9 (C1), 13.6 (C6); Anal. Calc. for C_23_H_19_BrClN_5_O_2_S (544.85); C, 50.70; H, 3.52; N, 12.85. Found C, 50.66; H, 3.59; N, 12.73.

Spirooxindole derivative, **9f**.


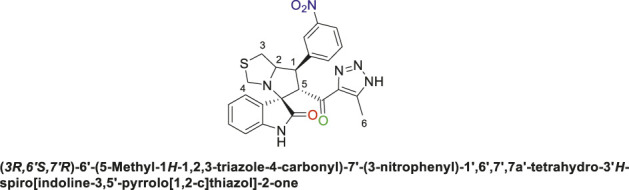


Pale-yellow solid with 73.5% yield; m.p. 201°C–202°C; ^1^H NMR (400 MHz, DMSO-_*d6*_) *δ* 15.20 (s, 1H, NH), 10.41 (s, 1H, NHC = O), 8.33 (s, 1H), 8.13 (dd, *J* = 8.1, 2.2 Hz, 1H), 7.99 (d, *J* = 7.5 Hz, 1H), 7.68 (p, *J* = 8.2, 7.5 Hz, 1H), 7.16 (s, 1H), 7.06 ((d, *J* = 8.8 Hz, 1H), 6.93–6.79 (m, 1H), 6.54 (d, *J* = 7.8 Hz, 1H) (8 aromatic protons), 4.90 (s, 1H, H-C5), 4.25 (s, 1H, H-C2), 4.12 (d, *J* = 10.3 Hz, 1H, H-C1), 3.67 (s, 1H, H-C4), 3.40 (s, 1H, H-C4), 3.14–2.84 (m, 2H, 2H-C3), 2.01 (s, 3H, 3H-C6); ^13^C NMR (126 MHz, DMSO-_*d6*_) *δ* 191.3 (C=O), 179.0 (-NHC = O), 148.5, 142.8, 142.0, 135.5, 130.9, 130.3, 127.5, 124.0, 123.3, 122.8, 121.4, 110.0 (Aromatic Carbons), 74.3 (shared C), 72.6 (C2), 65.0 (C4), 52.2 (C5), 48.8(C3), 35.3 (C1), 22.6 (C6); Anal. Calc. for C_23_H_20_N_6_O_4_S (476.51); C, 57.97; H, 4.23; N, 17.64. Found C, 58.09; H, 4.20; N, 17.77.

Spirooxindole derivative, **9g**.


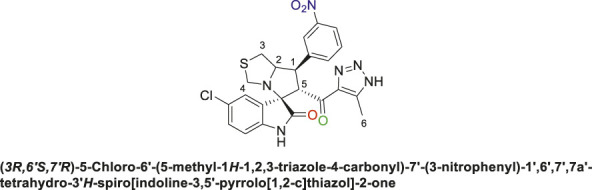


Off-white solid with 71% yield; m.p. 173°C–175°C; ^1^H NMR (400 MHz, DMSO-_*d6*_) *δ* 15.28 (s, 1H, NH), 10.55 (s, 1H, NHC = O), 8.36 (s, 1H), 8.22–8.11 (m, 1H), 8.11–7.93 (m, 1H), 7.69 (q, *J* = 8.0 Hz, 1H), 7.18 (q, *J* = 9.6, 8.6 Hz, 2H), 6.53 (dd, *J* = 19.4, 8.6 Hz, 1H), 4.89 (s, 1H, H-C5), 4.22 (d, *J* = 8.8 Hz, 1H, H-C2), 4.11 (d, *J* = 9.5 Hz, 1H, H-C1), 3.66 (d, *J* = 16.9 Hz, 1H, H-C4), 3.41 (s, 1H, H-C4), 3.00 (d, *J* = 5.5 Hz, 2H, 2H-C3), 2.17–1.98 (s, 3H, 3H-C6), ^13^C NMR (126 MHz, DMSO-_*d6*_) *δ* 191.1 (C=O), 179.0 (-NHC = O), 148.5, 142.2, 141.8, 141.5, 135.5, 130.9, 130.2, 127.4, 125.7, 125.7, 123.4, 122.9, 111.5 (Aromatic Carbons), 74.3 (shared C), 73.1 (C2), 64.7 (C4), 52.9 (C5), 49.1 (C3), 35.7) (C1), 22.5 (C6); Anal. Calc. for C_23_H_19_ClN_6_O_4_S (510.95); C, 54.07; H, 3.75; N, 16.45. Found C, 54.17; H, 3.88; N, 16.31.

Spirooxindole derivative, **9h**.


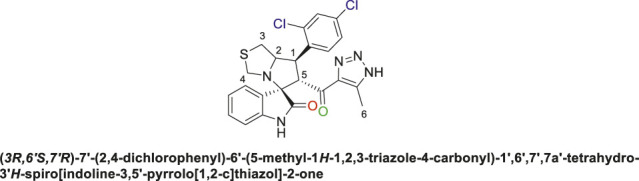


Pale-yellow precipitate with 91.9% chemical yield; m.p. 203°C–206°C; ^1^H NMR (400 MHz, DMSO-_*d6*_) *δ* 15.11 (s, 1H, NH), 10.41 (s, 1H, NHC = O), 7.80 (s, 1H), 7.62 (s, 1H), 7.51 (s, 1H), 7.09 (d, *J* = 19.8 Hz, 2H), 6.85 (s, 1H), 6.53 (s, 1H) (7 Aromatic protons), 4.88 (s, 1H, H-C5), 4.54 (s, 1H, H-C2), 4.33 (s, 1H, H-C1), 4.06 (s, 1H, H-C4), 3.72 (s, 1H, H-C4), 3.06 (s, 1H, H-C3), 2.90 (s, 1H, H-C3), 2.01 (s, 3H, 3H-C6), ^13^C NMR (101 MHz, DMSO-_*d6*_) *δ* 191.3 (C=O), 178.8 (NHC = O), 143.1, 142.1, 137.6, 134.9, 132.7, 131.0, 130.2, 129.4, 128.6, 127.4, 123.9, 121.3, 109.8 (Aromatic Carbons), 75.2 (shared spiro C), 72.7 (C2), 64.3 (C4), 52.5 (C5), 44.0 (C3), 35.4 (C1), 14.5 (C6); Anal. Calc. for C_23_H_19_Cl_2_N_5_O_2_S (500.40); C, 55.21; H, 3.83; N, 14.00. Found C, 55.19; H, 3.77; N, 14.11.

Spirooxindole derivative, **9i**.


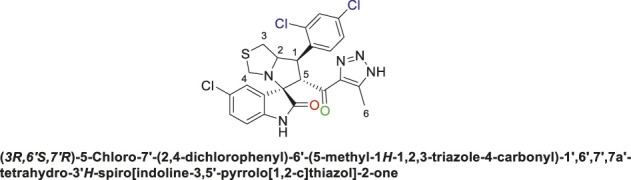


Off-white solid in 82.3% yield; m.p. 188°C–190°C; ^1^H NMR (400 MHz, DMSO-_*d6*_) *δ* 15.30 (s, 1H, NH), 10.63 (s, 1H, NHC = O), 7.82 (t, *J* = 8.8 Hz, 1H), 7.62 (s, 1H), 7.51 (d, *J* = 10.3 Hz, 1H), 7.13 (d, *J* = 8.1 Hz, 1H), 7.07 (d, *J* = 16.1 Hz, 1H), 6.58 (s, 1H) (6 Aromatic protons), 4.86–4.92 (d, *J* = 9.5 Hz, 1H, H-C5), 4.50 (d, *J* = 13.9 Hz, 1H, H-C2), 4.08 ((d, *J* = 12.5 Hz, 1H, H-C1), 3.73 (d, *J* = 9.4 Hz, 1H, C4), 3.40 (d, *J* = 3.3 Hz, 1H, H-C4), 3.09 (d, *J* = 16.9 Hz, 1H, H-C3), 2.87 (dd, *J* = 10.7, 5.8 Hz, 1H, H-C3), 2.04 (s, 3H, 3H-C6), ^13^C NMR (101 MHz, DMSO-_*d6*_) *δ* 191.2 (C=O), 178.5 (-NHC = O), 142.3, 141.7, 138.0, 137.7, 134.8, 132.8, 131.2, 130.2, 129.5, 128.6, 127.2, 126.1, 125.3, 111.3 (Aromatic Carbons), 75.3 (d, *J* = 14.2 Hz, Shared C), 72.7 (d, *J* = 23.4 Hz, C2), 64.5 (C4), 43.8 (C5), 35.5 (C3), 29.6 (C1), 13.8 (C6); Anal. Calc. for C_23_H_18_Cl_3_N_5_O_2_S (534.84); C, 51.65; H, 3.39; N, 13.09. Found C, 51.77; H, 3.42; N, 13.00.

### 2.2 Computational details

The *ω*B97X-D functional, (Chai and Head-Gordon, 2008), together with the standard 6-311G (d,p) basis set, (Hehre et al., 1986), which includes d-type polarization for second row elements and p-type polarization functions for hydrogen atoms, were used in this MEDT study. The TSs were characterized by the presence of only one imaginary frequency. The solvent effects of methanol were considered by fully optimizing the gas-phase structures at the same computational level using the polarizable continuum model (PCM) (Tomasi and Persico, 1994; Simkin, 1995) Values of *ω*B97X-D/6-311G (d,p) enthalpies, entropies and Gibbs free energies in methanol were calculated with standard statistical thermodynamics at 337.8 K and 1 atm, (Hehre et al., 1986), by PCM frequency calculations at the solvent optimized structures. The GEDT values were computed by using the equation GEDT (f) = Σq_f_, where q represents the natural charges of the atoms belonging to one of the two frameworks (f) at the TS geometries. (Domingo, 2014; Reed et al., 1985; Reed et al., 1988). Global and local CDFT indices were calculated by using the equations given in reference. (Parr and Yang, 1989; Domingo et al., 2016). The Gaussian 16 suite of programs was used to perform the calculations. (Frisch et al., 2016). Molecular geometries were visualized by using the GaussView program. (Dennington et al., 2016).

### 2.3 Biology

#### 2.3.1 Molecular docking study

The protocol for the molecular docking study is available in the Supplementary Material.

#### 2.3.2 ADME properties

In this study, the SwissADME web server (http://www.swissadme.ch/) was used to calculate the ADME (Absorption, Distribution, Metabolism, and Excretion) parameters for the most active compounds in the series. The SMILES (Simplified Molecular Input Line Entry System) representations of the compounds were submitted to the SwissADME server to obtain various pharmacokinetic properties, including lipophilicity, pharmacokinetics, drug-likeness, water solubility, and synthetic accessibility.

#### 2.3.3 Molecular dynamic simulation

Additionally, a brief production run of 100 ns using AMBER22 was carried out to examine the dynamic stability of all three compounds **6b**, **6c**, and **9h** in the EGFR active site. (Case et al., 2023). The prior specified procedures for system preparation, including minimization and equilibrium, were followed (Chhikara et al., 2020). Trajectory analysis was conducted using the AMBER suite’s integrated CPPTRAJ module (Roe DR, Cheatham III TE. PTRAJ and CPPTRAJ: software for processing and analysis of molecular dynamics trajectory data. (Roe and Cheatham, 2013).

#### 2.3.4 Anti-cancer reactivity

The protocol for the initial screening and cell viability by MTT assay is provided in the Supplementary Material.

## 3 Results and discussion

### 3.1 Chemistry

**SCHEME 1:**
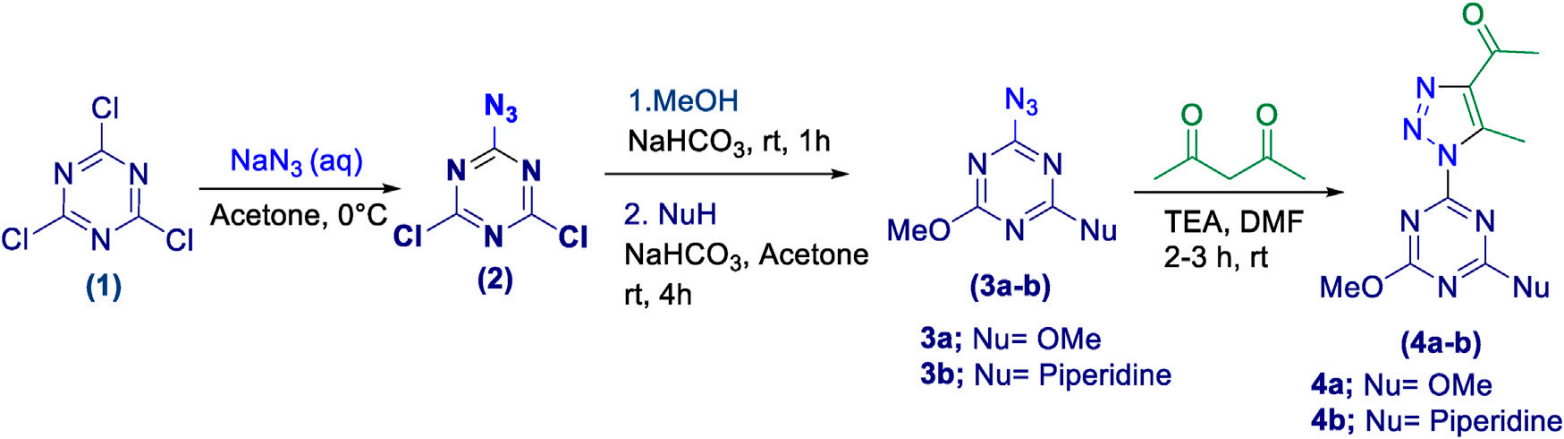
Synthetic route of triazolyl-*s*-triazine derivatives **(4a-b)**.

Initially, a multistep synthetic methodology was employed, starting with cyanuric chloride **(1)** as the triazine scaffold to synthesize a novel series of spirooxindole derivatives incorporating a triazolyl-*s*-triazine skeleton. The first step involved the reaction of cyanuric chloride **(1)** with sodium azide to obtain azido-*s*-triazine **(2)**, following the reported method. (Sharma et al., 2019; Sharma et al., 2020). Subsequently, the two chlorine atoms of **(2)** were sequentially substituted by nucleophiles, such as methoxy and piperidinyl substituents, through a two-step synthetic route. This led to the synthesis of the corresponding disubstituted azido-*s*-triazine derivatives **(3a-b)** according to the reported method with a slight modification. (Mylari et al., 2003). The subsequent step involved a 32CA reaction of **(3a-b)** with acetylacetone to obtain the corresponding triazolyl disubstituted-*s*-triazine intermediates **(4a-b)**, following the reported method, as outlined in Scheme 1. (Mylari et al., 2003).

**SCHEME 2:**
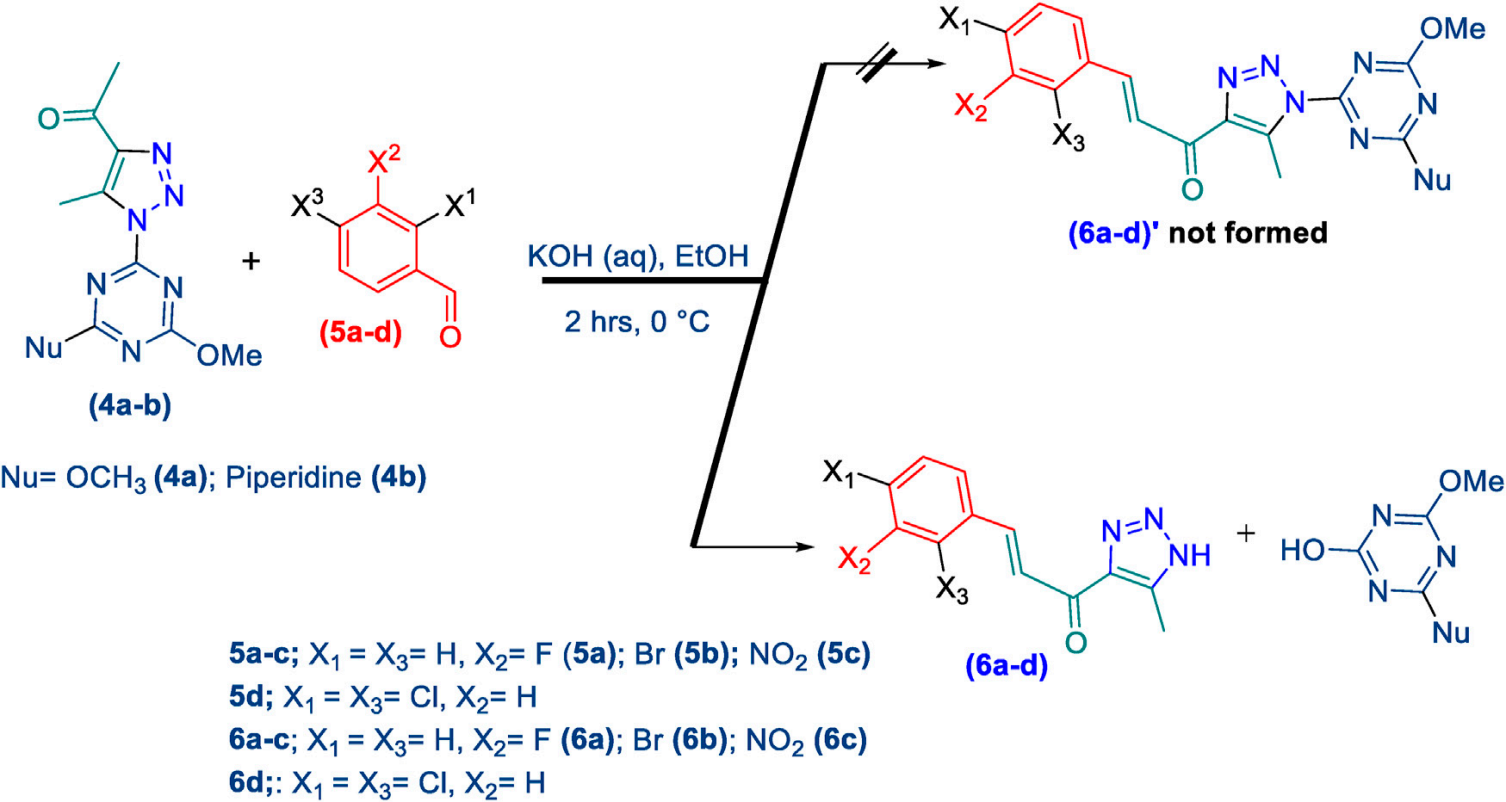
Synthesis of 1,2,3-triazole chalcones **(6a-d)**.

Thereafter, hybrids **(4a-b)** underwent a *Claisen-Schmidt condensation (cross-aldol condensation)* reaction with several benzaldehyde derivatives **(5a-d)** in the presence of aqueous potassium hydroxide-ethanol, as per the reported methods with slight modifications, to obtain the target chalcones. (Basedia et al., 2011; Islam et al., 2022). However, the reaction did not progress as anticipated, encountering two specific challenges. The first challenge was the formation of a mixture of byproducts, as detected by TLC (ethyl acetate/n-hexane 4:6), which led to a notable decrease in the chemical yield. To address this issue, various conditions were tested, including temperature, the concentration of KOH, and the sequential order of adding the materials. Optimal conditions were achieved when triazolyl disubstituted-*s*-triazine derivatives **(4a-b)** were initially treated with 7% (w/v) aqueous KOH under gentle heating to ensure complete formation of the corresponding enolate anion. This enolate anion then underwent *Claisen-S. condensation (cross-aldol condensation)* with benzaldehyde derivatives **(5a-d)**, as shown in Scheme 2. Furthermore, the reaction’s progress was controlled thermally by immersing the mixture in an ice bath.

The second challenge arose when the previously optimized conditions were applied, by reacting the two triazolyl disubstituted-*s*-triazine derivatives **(4a-b)** with four benzaldehyde derivatives **(5a-d)**. As expected, the goal was to obtain eight different chalcones. However, the spectroscopic data showed the formation of only four chalcones. This observation supported the assumption that the identity of the new chalcone depended solely on the type of benzaldehyde used, regardless of which triazolyl disubstituted-*s*-triazine derivative **(4a** or **4b)** was employed. A possible explanation is that cleavage of the triazine ring occurred due to the hydrolysis in the strongly basic medium. For Instance, the ^1^H-NMR spectrum of the chalcone (*E*)-3-(2,4-dichlorophenyl)-1-(5-methyl-1*H*-1,2,3-triazol-4-yl)prop-2-en-1-one **(6d) (** Supplementary Figure S6 **)**, derived from the methoxy-piperidinyl triazolyl-*s*-triazine derivative **(4b)** did not show any signals in the range of *δ* 3.6–3.8 ppm, where the methoxy group (attached to triazine ring) or the methylene protons (adjacent to piperidine nitrogen) are expected to appear. This observation reinforces the hypothesis that the *s*-triazine ring was cleaved from the rest of the chalcone skeleton. Interestingly, the same ^1^H-NMR signals were observed for the chalcone prepared from the dimethoxy-triazolyl-*s*-triazine derivative **(4a)**, confirming the conclusion that both chalcones are identical due to the hydrolysis-cleavage of the *s*-triazine moiety. Additionally, the ^13^C-NMR spectrum of chalcone **(6d) (** Supplementary Figure S6 **)**, prepared from either **(4a)** or **(4b)**, showed remarkable similarity, with no signals corresponding to the s-triazine, methoxy, and piperidine carbons. This unequivocally confirms the previous conclusion. We attempted to use various bases to bypass the hydrolysis step but were unsuccessful. Weaker bases, such as K_2_CO_3_, were unable to produce the chalcone at all. In contrast, the stronger base NaOH caused cleavage of the triazine scaffold even at very low concentrations, demonstrating its effectiveness as a labile group. On the other hand, it was challenging to precisely identify the vicinal protons of the chalcones **(4a-d)** based on the ^1^H-NMR spectrum, due to their signals overlapping with the aromatic protons. However, it is highly probable that the peaks at 7.71 ppm (d, *J* = 10.9 Hz, 1H) and 7.56 ppm (d, *J* = 12.6 Hz, 2H) in chalcone **(6a)**, as an example, correspond to vicinal protons, as these are the only signals with *J* values in the range of 11–12 Hz, which is consistent with trans-vicinal protons. It is evident that the four aromatic protons and the two vicinal protons are all clearly present in the range between 7.2 and 7.9 ppm, as illustrated in the spectrum (Supplementary Figure S3).

**SCHEME 3:**
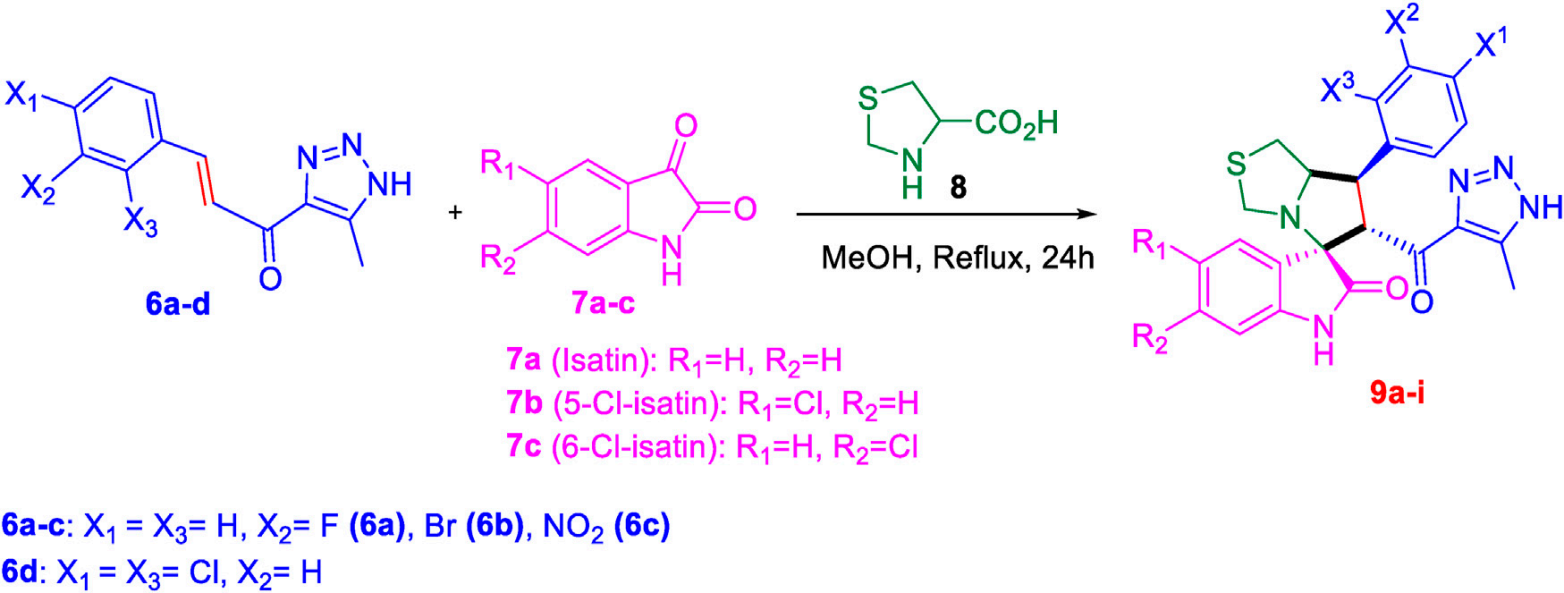
Synthetic approach of spirooxindole derivatives **(9a-i)**.

Subsequently, the chalcones **(6a-d)** were subjected to a one-pot multicomponent reaction with isatin derivatives **(7a-c)** and (s)-thiazolidine amino acid **(8)** to synthesize a novel series of 12 spirooxindole derivatives **(9a-i)** following the reported methods. (Altowyan et al., 2019; Altowyan et al., 2020; Al-Majid et al., 2019; Badria et al., 2019; Yasmine et al., 2021). The optimal reaction conditions were established by mixing 1 equivalent of each reactant in methanol, with stirring for 12–24 h under reflux, as shown in Scheme 3.

The structural elucidation of the derivatives **(9a-i)** was carried out using different characterization techniques, such as NMR (^1^H- and ^13^C-), IR, and Elemental analysis. The obtained results were in full agreement with the proposed structures. For example, the ^1^H-NMR spectrum of compound (9c) (Supplementary Figure S9) showed a singlet signal for the NH proton at 10.51 ppm, whereas the seven aromatic protons appeared in the range of 6.53–7.37 ppm. The methine proton at C-5 appeared as a doublet at 4.84 ppm (J = 10.7 Hz, vicinal coupling), while the methine protons at C-2 and C-1 resonated at 4.12 ppm and 3.88 ppm, respectively. The protons of the two magnetically inequivalent methylene groups at C-4 and C-3 were observed at δ 3.61–3.36 (two protons) and δ 2.88–2.98 (two protons), respectively. The multiplet in the range of 1.81–2.11 ppm corresponded to the three methyl protons at C-6. Additionally, the three methine carbons at C-2, C-5, and C-1 appear at *δ* 71.81, 51.65, and 35.19 respectively. The (Yuenyongsawad et al., 2013)C-NMR spectrum (Supplementary Figure S9) showed one signal at *δ* 191.25 corresponding to the carbonyl-carbon adjacent to the triazole moiety, and another signal *δ* 178.71 characteristic of the oxindole carbonyl-carbon. The fourteen signals in the range of *δ* 114.45–163.78 corresponded to the aromatic carbons. The signal at *δ* 74.32 was characteristic of the quaternary shared carbon, while the two signals at *δ* 64.96 and 35.19 were assigned to the methylene carbons, C-2 and C-3, respectively. The methyl carbon at C-6 appeared at *δ* 8.67.

It is estimated that compounds **(9a-i)** were formed through a [3 + 2] cycloaddition reaction, following an *ortho/endo* pathway, in agreement with similar structural frameworks. (Shahidul Islam et al., 2021; Saleh Altowyan et al., 2022; Domingo et al., 2022). This will be further explored in the subsequent MEDT study.

### 3.2 Computational studies

#### 3.2.1 MEDT study of the 32CA reaction of azomethine ylide (AY, 10m) with ethylene (6m)

**SCHEME 4:**
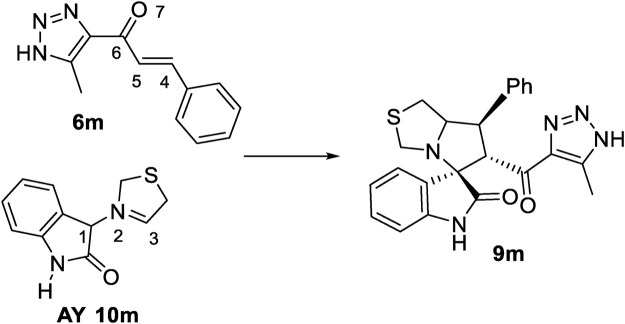
Synthetic route of spirooxindole **(9m)** via 32CA reaction.

This section presents a theoretical investigation of the 32CA reaction between the azomethine ylide (AY**, 10m**), R_1_ = R_2_ = H, and ethylene (**6m)**, X_1_ = X_2_ = X_3_ = H, yielding spirooxindole (**9m)**. The study was conducted using Molecular Electron Density Theory (MEDT) (Domingo, 2016) to explore the characteristics of these 32CA reactions (see Scheme 4).

#### 3.2.2 Conceptual DFT analysis of the reagents

The reactivity indices, as outlined in Conceptual DFT (CDFT) (Parr and Yang, 1989; Domingo et al., 2016) have proven to be significant tools for comprehending the reactivity in polar reactions. (Domingo and Ríos-Gutiérrez, 2022). The primary global reactivity indices, which include electronic chemical potential *μ*, chemical hardness *η*, electrophilicity ω, and nucleophilicity *N* indices for AY **(10m)** and ethylene **(6m)** were compiled in Table 1.

**TABLE 1:** B3LYP/6-31G(d) values for electronic chemical potential *μ*, chemical hardness *η*, electrophilicity ω and nucleophilicity *N* indices, expressed in eV, for AY **(10m)** and ethylene **(6m)**.

	*μ*	*η*	ω	*N*
ethylene **(6m)**	−4.07	4.29	1.93	2.91
AY **(10m)**	−3.11	3.25	1.48	4.39

The electronic chemical potential *μ* (Parr and Pearson, 1983) of AY **(10m)** was −3.11 eV, higher than that of ethylene **(6m)** at −4.07 eV. This difference suggested that in a polar 32CA reaction, there would be a global electron density transfer (GEDT) (Domingo, 2014) from AY **(10m)** to ethylene **(6m)**, indicating a forward electron density flux (FEDF) classification for the reaction. (Doming and Ríos-Gutiérrez, 2023).

AY **(10m)** exhibited an electrophilicity ω index (Parr et al., 1999) of 1.48 eV, categorizing it as a moderate electrophile on the electrophilicity scale, (Domingo and Ríos-Gutiérrez, 2022), and a nucleophilicity *N* index (Domingo et al., 2008a) of 4.39 eV, which qualified it as a strong nucleophile within the same scale. (Domingo and Ríos-Gutiérrez, 2022). With a nucleophilicity value exceeding 4.0 eV, AY **(10m)** was classified as a supernucleophile. (Domingo and Ríos-Gutiérrez, 2022).

The electrophilicity ω index of ethylene **(6m)** was 1.92 eV, classifying it as a strong electrophile according to the electrophilicity scale. (Domingo and Ríos-Gutiérrez, 2022). Conversely, **6m** exhibited a nucleophilicity *N* index of 2.91 eV, categorizing it as a moderate nucleophile on the nucleophilicity scale. (Domingo and Ríos-Gutiérrez, 2022).

The super nucleophilic nature of AY **(10m)**, combined with the strong electrophilic character of ethylene **(6m)**, implied that their subsequent 32CA reaction would exhibit a significant polar character, classifying it as FEDF. (Doming and Ríos-Gutiérrez, 2023). Such distinct polar characteristics further enhanced the already favorable *pdr-type* 32CA reaction.

**FIGURE 2:**
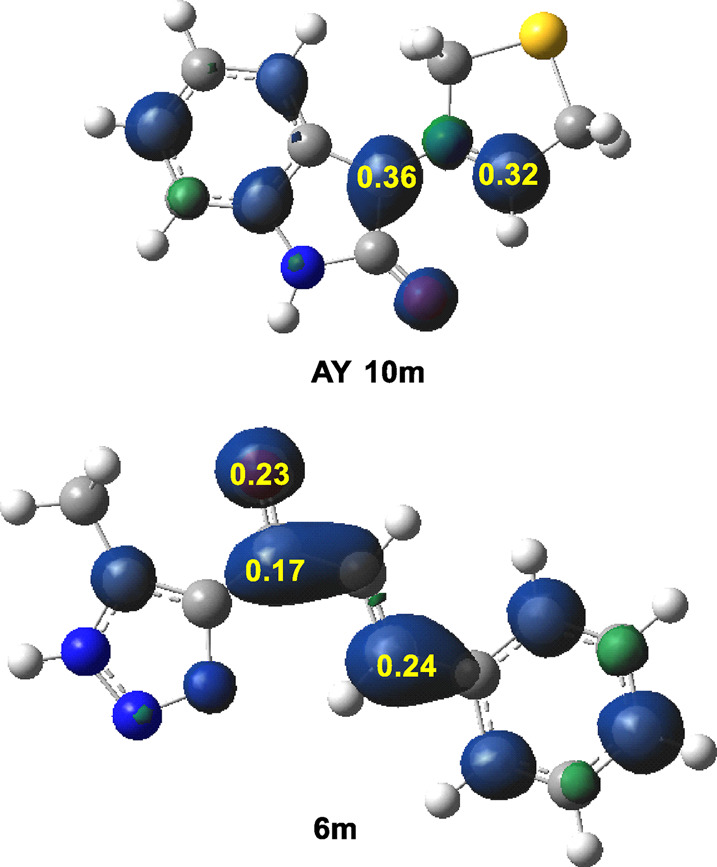
B3LYP/6-31G(d) analysis of the nucleophilic P_k_^−^ Parr functions of AY **(10m)** and the electrophilic P_k_^+^ Parr functions of ethylene **(6m)**.

In a polar reaction with asymmetric species, the preferred reaction pathway generally features two-center interaction between the most nucleophilic and the most electrophilic centers. (Aurell et al., 2004). Numerous studies have highlighted that one of the most precise and informative methods for examining local reactivity in polar and ionic processes (Domingo and Ríos-Gutiérrez, 2022) is the analysis of the electrophilic P_k_^+^ and nucleophilic P_k_^−^ Parr functions. (Domingo et al., 2013). These functions result from the excess of spin electron density through global electron density transfer (GEDT). (Domingo, 2014). Consequently, considering the electronic characteristics of the reactants, the nucleophilic P_k_^−^ Parr functions of AY **(10m)** and the electrophilic P_k_^+^ Parr functions of ethylene **(6m)** were examined as depicted in (Figure 2).

In AY **(10m)**, the nucleophilic activation of the C1 and C3 carbons was indicated by P_k_^−^ values of 0.36 and 0.32, respectively; with C1 carbon showing a slightly higher activation than the C3 carbon. Note that the nitrogen atom was deactivated. This feature suggested a low regioselectivity based only on electronic activation. Conversely, in the electrophilic ethylene **(6m)**, the *β*-conjugated C4 carbon emerged as the most electrophilically activated center with a P_k_^+^ value of 0.24. It was important to note that the β-conjugated C4 carbon exhibited twice the electrophilic activation compared to the carbonyl C6 carbon. Therefore, the preferred two-center interactions at the transition state (TS) were likely to occur between the β-conjugated C4 carbon of ethylene **(6m)** and either the C1 or C3 carbon of AY **(10m)**.

#### 3.2.3 Study of the 32CA reaction of AY (10m) with ethylene (6m)

**SCHEME 5:**
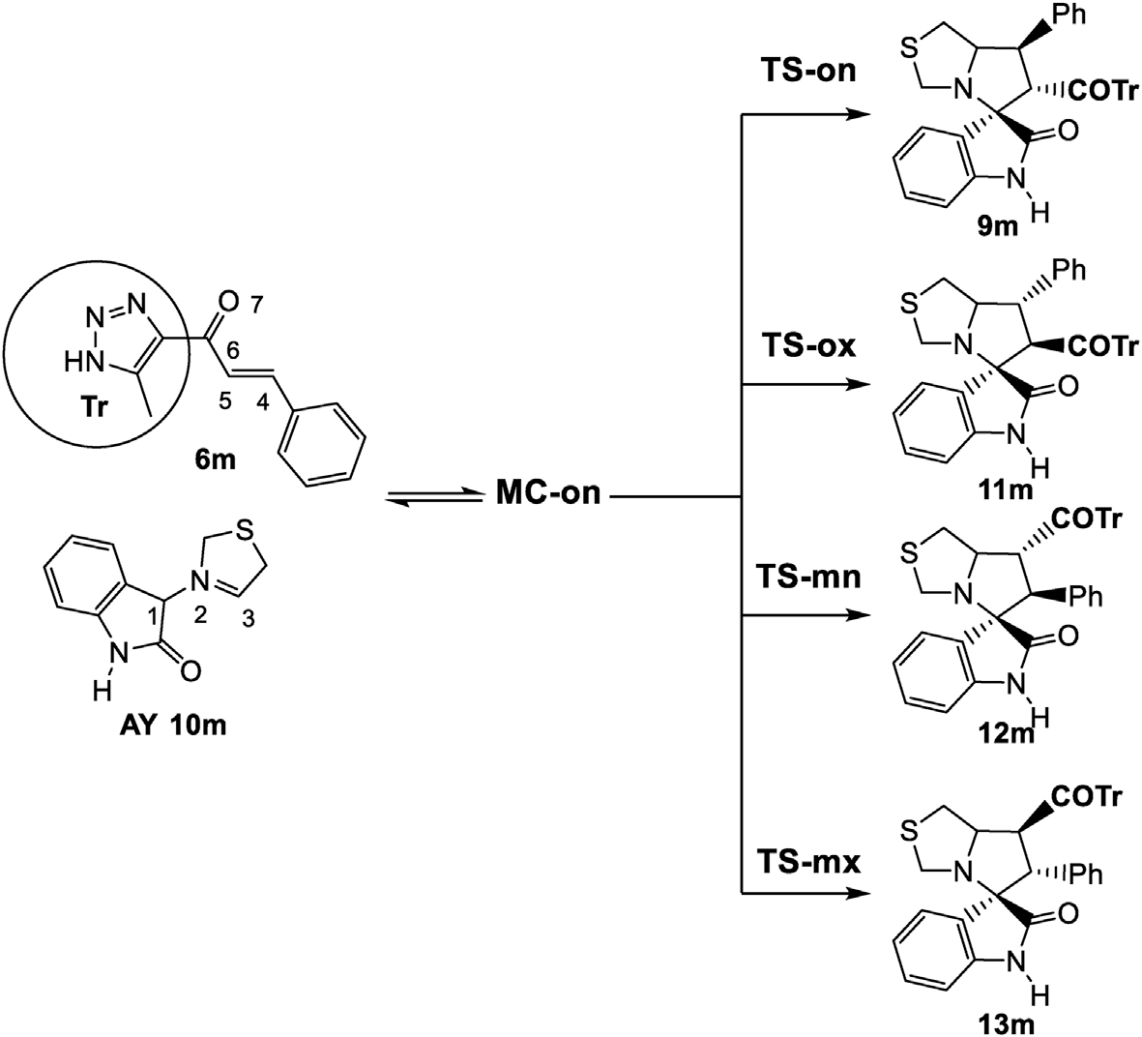
32CA reaction of AY **(10m)** with the ethylene **(6m)**.

According to the non-symmetry of both reactants, there are feasible options for two pairs of *endo* and *exo* stereoisomeric pathways, as well as two pairs of *ortho* and *meta* regioisomeric pathways. These four competitive pathways were investigated as illustrated in (Scheme 5). The analysis of the stationary points identified along the four reaction paths suggested that this 32CA reaction proceeded via a one-step mechanism. The relative enthalpies and Gibbs free energies calculated using *ω*B97X-D/6-311G (d,p) were shown in (Table 2), with complete thermodynamic data available in (Supplementary Table S2) of the Supplementary Material.

**TABLE 2:** *ω*B97X-D/6-311G (d,p) relative enthalpies (ΔH in kcal·mol^−1^), entropies, (ΔS in cal·mol^−1^K^−1^), and Gibbs free energies (ΔG in kcal·mol^−1^), concerning the separated reagents, computed at 337.85 K and 1 atm in methanol, for the stationary points involved in the 32CA reaction of AY **(10m)** with ethylene **(6m)**.

	ΔH	ΔS	ΔG
**MC-on**	−19.8	−43.5	−5.1
**TS-on**	−11.9	−54.2	6.4
**TS-ox**	−5.7	−52.7	12.1
**TS-mn**	−7.7	−49.6	9.0
**TS-mx**	−5.4	−48.6	11.0
**9m**	−44.1	−52.6	−26.3
**11m**	−49.0	−49.3	−32.4
**12m**	−47.4	−51.0	−30.2
**13m**	−50.9	−49.2	−34.3

Several molecular complexes (MCs) characterized by weak intermolecular interactions binding the two reagents were identified. Among these complexes, only the most stable, referred to as **MC-on**, was chosen as the energy reference, as all of them existed in thermodynamic equilibrium. At this molecular complex (MC), the distance between the two frameworks was approximately 3.2 Å. **MC-on** was observed to be 18.6 kcal·mol^−1^ lower in energy compared to the separated reagents as detailed in (Table 2). The significant stabilization observed in this polar molecular complex allowed it to be classified as an electron density transfer complex (EDTC). (Org. Biomol. Chem., 2019, 17, 6478). Several significant conclusions could be drawn from the relative enthalpies detailed in Table 2: i) The optimal transition state **(TS-on)** was identified at 11.9 kcal·mol^−1^ below the energy of the separated reagents. However, when considering the formation of **MC-on**, the activation enthalpy turned positive, increasing by 7.9 kcal·mol^−1^; ii) this 32CA reaction exhibited complete *endo* stereoselectivity since **TS-ox** is situated 6.2 kcal·mol^−1^ higher than **TS-on**; iii) this 32CA reaction exhibited complete *ortho* regioselectivity because the transition state **TS-mn** was 4.2 kcal·mol^−1^ higher in energy than **TS-on**, and iv) the 32CA reaction exhibited significant exothermicity due to spirooxindole **(9m)** being energetically 44.1 kcal·mol^−1^ lower than the separated reactants. Hence, employing kinetic control facilitated the formation of spirooxindole **(9m)**.

**FIGURE 3:**
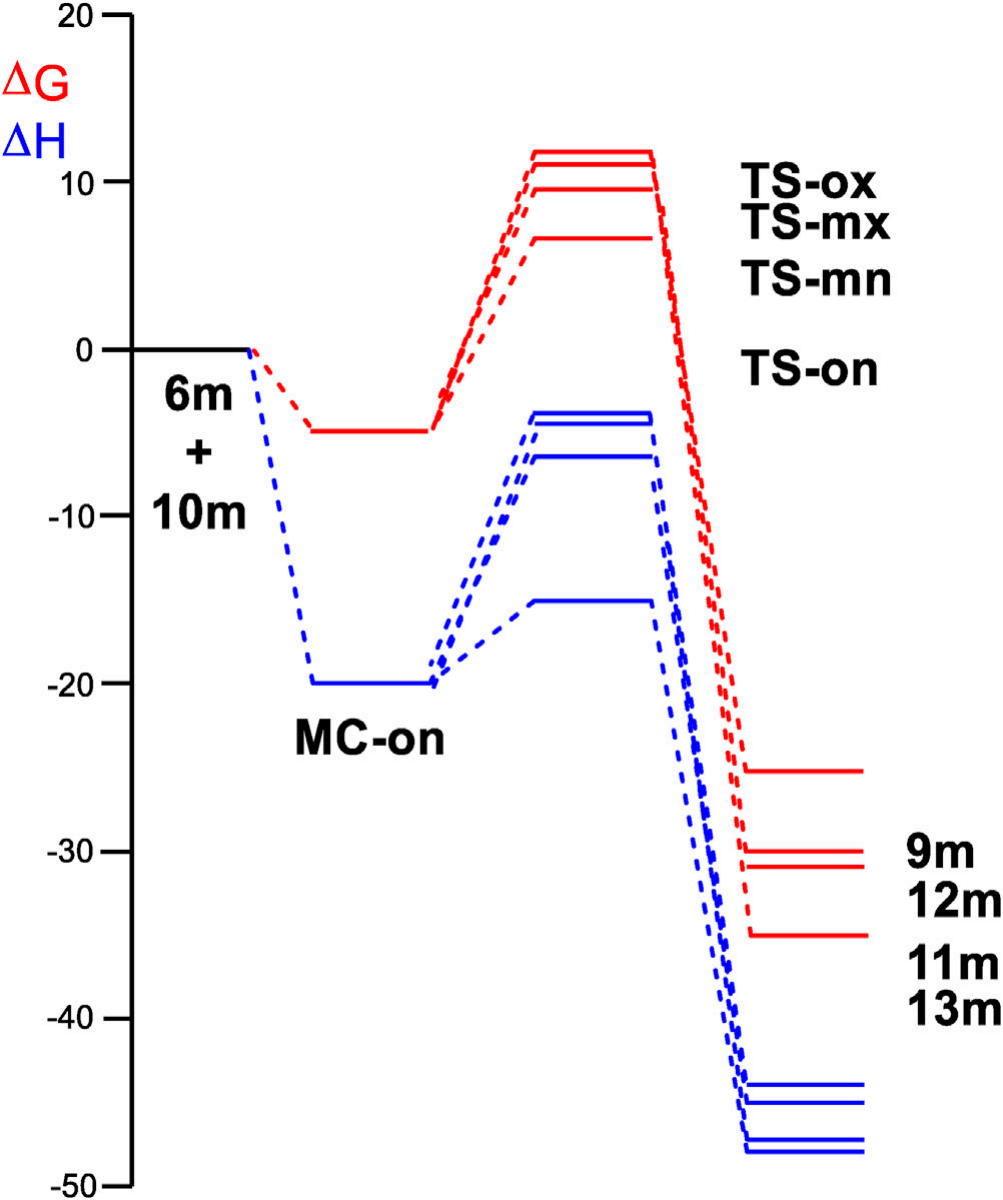
Enthalpy (ΔH, represented in blue) and Gibbs free energy (ΔG, represented in red) profiles using *ω*B97X-D/6-311G (d,p), expressed in kcal·mol^–1^, computed in methanol at 65°C for the 32CA reaction involving AY **(10m)** and ethylene **(6m)**.

In (Figure 3) the enthalpy and Gibbs free energy profiles for the four competing reaction pathways were depicted. Incorporating thermal corrections and entropies into enthalpies resulted in a rise in relative Gibbs free energies by 14.7 to 18.3 kcal·mol^−1^. This increase was attributed to the unfavorable entropies associated with this bimolecular reaction, which ranged from −43.5 to −54.2 kcal·mol^−1^·K^−1^. The exergonic behavior associated with the formation of **MC-on,** identified by a value of 5.0 kcal·mol^−1^ was consistent with the predictions of an EDTC. Meanwhile, the activation Gibbs free energy for the 32CA reaction of AY **(10m)** with ethylene **(6m)** through **TS-on** was calculated at 6.4 kcal·mol^−1^. Additionally, the creation of spirooxindole **(10m)** was also exergonic, characterized by a substantial energy release of 26.3 kcal·mol^−1^. Reflecting on the activation Gibbs free energies, this 32CA reaction exhibited complete *endo* stereoselectivity and *ortho* regioselectivity, as **TS-ox** and **TS-mn** were positioned 5.7 and 2.6 kcal·mol^−1^, respectively, above **TS-on** as depicted in (Figure 3).

**FIGURE 4:**
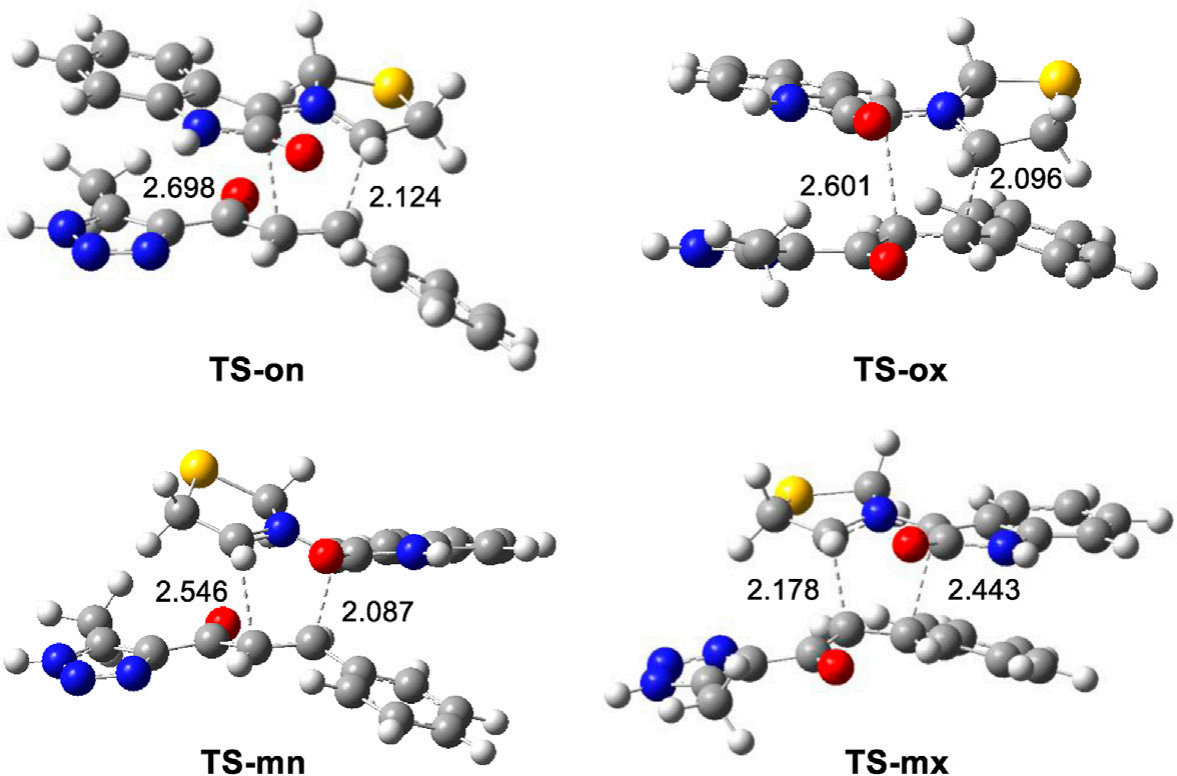
32CA reaction between AY **(10m)** and ethylene **(6m)**. Distances are measured in angstroms, Å.

The geometries of the four transition states (TSs) optimized in methanol were illustrated in (Figure 4). In these four transition states, the C−C distances between the four interacting carbons showed that, except for the least favorable **TS-mx**, the other three TSs were associated with asynchronous C−C single bond formation processes. Notably, the shorter C−C distance in these processes involved the participation of the highly electrophilic *β*-conjugated C4 carbon of ethylene **(6e)**. The C−C distances at the most favorable **TS-on**, which were 2.124 Å (C3−C4) and 2.697 Å (C1−C5) revealed a highly asynchronous C-C single bond formation process at this transition. The results of the intrinsic reaction coordinate analysis (Fukui, 1970) for the highly asynchronous **TS-on** revealed that the 32CA reaction followed a non-concerted, *two-stage*, *one-step* mechanism. (Domingo et al., 2008b). In this process, the formation of the second C1−C5 single bond only began after the first C3−C4 single bond was completely formed.

Finally, the quantification of the polar character of this 32CA reaction was facilitated by analyzing GEDT (Domingo, 2014) at the most favorable **TS-on**. Values below 0.05 e suggested non-polar processes, while those exceeding 0.20 e indicated polar processes. The high GEDT value at **TS-on**, which measures 0.23 e, indicates the highly polar character of this 32CA reaction. This outcome stemmed from the supernucleophilic character of AY **(10m)** and the strong electrophilic nature of ethylene **(6m)**, detailed in (Table 1). Furthermore, this 32CA reaction was categorized as FEDF based on the flux from AY **(10m)** to ethylene **(6m)**, consistent with the analysis of CDFT indices.

### 3.3 Biology

#### 3.3.1 *In vitro* antiproliferative activity

**FIGURE 5:**
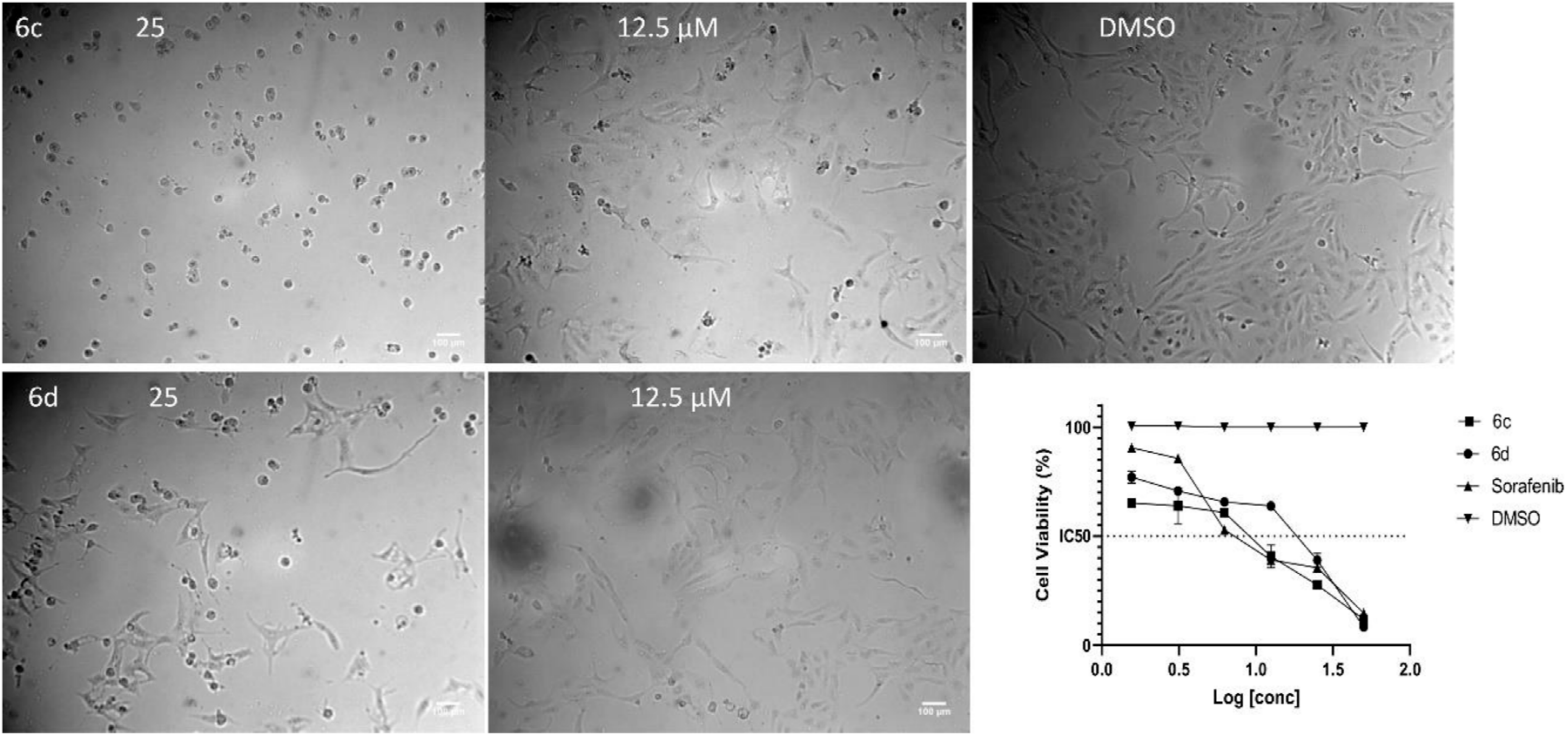
Representative images for the activity of chalcone derivatives **6c** and **6d** against HepG2. Dead cells at 25 µM and the activity was decreased at 12.5 µM after 48 h of incubation with the compounds. The IC_50_ was calculated by GraphPad Prism software and Sorafenib was used as a positive control for the MTT assay.

The analysis of cell viability (%) presented in Table 3 indicated that the compounds exhibited cytotoxicity at a tested concentration of 50 µM after a 48-h incubation period. The concentration at which 50% of cells were killed (IC_50_) was determined through a series of 6-point serial dilutions (ranging from 50 to 1.56 µM) for each compound. A solution containing 0.5% DMSO functioned as the negative control, while Sorafenib acted as the positive control. Cell viability was measured using the MTT assay in this study. Prism Software was utilized for IC_50_ calculation, and the corresponding data were detailed in Table 4 and Figure 5.

**TABLE 3:** Cell viability (%) following the incubation of cells with 50 µM of the tested compounds.

Compound	MDA-MB-231	HepG2
Chalcones		
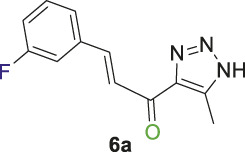	95.5 ± 1.53	73.4 ± 1.09
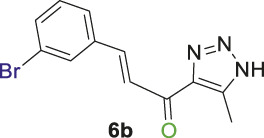	42.5 ± 0.54	26.9 ± 0.53
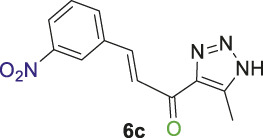	29.8 ± 0.14	14.5 ± 0.46
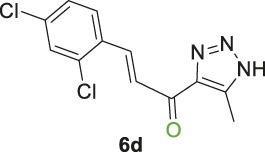	19.8 ± 0.78	10.7 ± 0.75
Spiro Derivatives		
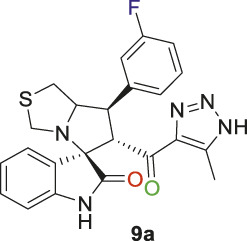	83.1 ± 1.36	40.7 ± 0.17
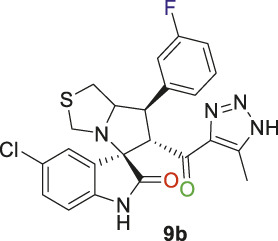	27.1 ± 0.82	23.0 ± 0.04
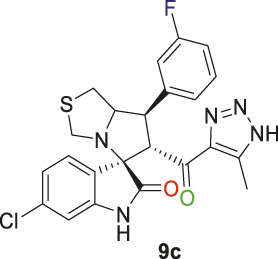	23.1 ± 0.73	11.4 ± 0.01
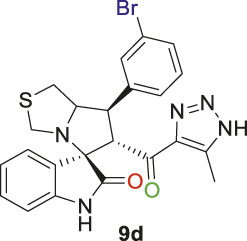	70.0 ± 1.05	73.4 ± 0.74
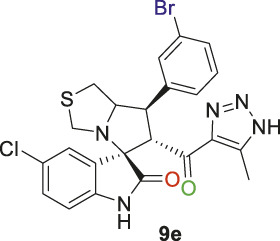	67.7 ± 0.41	77.9 ± 0.37
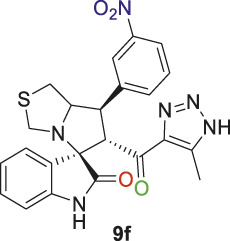	101.1 ± 5.53	69.9 ± 1.82
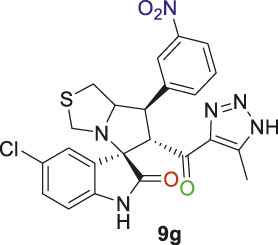	89.8 ± 2.08	82.3 ± 3.98
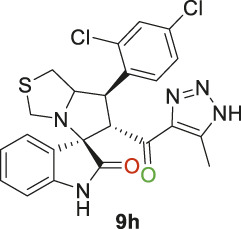	23.1 ± 0.43	8.4 ± 0.08
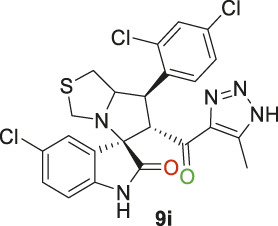	19.8 ± 0.52	7.7 ± 0.04

**TABLE 4:** IC_50_ (µM) for the most active compounds.

Compound	MDA-MB-231 IC_50_ (µM ± SD)	HepG2 IC_50_ (µM ± SD)
Chalcones		
6c	7.2 ± 0.56	7.5 ± 0.281
6d	11.1 ± 0.37	11 ± 0.282
Spiro Derivatives		
9b	31.3 ± 0.86	19.1 ± 0.978
9c	30.2 ± 1.60	24.2 ± 0.212
9h	16.8 ± 0.37	17 ± 0.131
9i	18.5 ± 0.74	13.5 ± 0.919
Sorafenib	9.98 ± 0.05	2.6 ± 0.01

The IC_50_ (µM) for the most active compounds were listed in Table 4. As shown, the most active chalcones, **6c** and **6d** exhibited similar reactivity for both cancer cell lines (MDA-MB-231 and HepG2). The chalcone-based compound **6c**, which incorporates nitro group as an electron-withdrawing moiety, demonstrated enhanced reactivity against the breast cancer cell line MDA-MB-231, with an IC_50_ of 7.2 ± 0.56 µM, outperforming the standard drug sorafenib, with an IC_50_ of 9.98 ± 0.05 µM. However, its effectiveness was lower in liver cancer (HepG2) cells, showing an IC_50_ of 7.5 ± 0.281 µM, compared to the reference drug’s IC_50_ of 2.6 ± 0.01 µM. Compound **6d** with 2,4-dicholrophenyl moiety showed less reactivity with a similar IC_50_ for both cell lines. The IC_50_ was determined for the most promising spirooxindoles compounds: **9b, 9c, 9h**, and **9i**. Spirooxindole **9b** incorporating 5-Cl-isatin and 3-Fluoro-phynyl substituent exhibited the lowest IC_50_ among the four hits against the breast cancer cell line (MDA-MB-231; IC_50_ = 31.3 ± 0.86 µM). The second most reactive compound, **9c**, features a spirooxindole scaffold with 6-chloro-isatin and a 3-fluorophenyl moieties, demonstrating an IC_50_ of 30.2 ± 1.60 µM against the breast cancer cell line MDA-MB-231. The third most reactive compound was **9i**, which contains a spirooxindole scaffold with 5-chloro-isatin and a 2,4-dichlorophenyl substituents, demonstrating an IC_50_ of 18.5 ± 0.74 µM against the MDA-MB-231 breast cancer cell line. The most active compound among the synthesized spirooxindoles was found to be **9h**, consisting of unsubstituted isatin and 2,4-dichlorophenyl moities, with an IC_50_ of 16.8 ± 0.37 µM in the same cell line. On the other hand, for liver cancer (HepG2) cells, the reactivity order was as follows: **9c** with an IC_50_ of 24.2 ± 0.212 µM, followed by **9b** at 19.1 ± 0.978 µM, then **9h** with 17 ± 0.131 µM, and the most reactive was **9i**, which displayed an IC_50_ of 13.5 ± 0.919 µM.

#### 3.3.2 Molecular docking study

**FIGURE 6:**
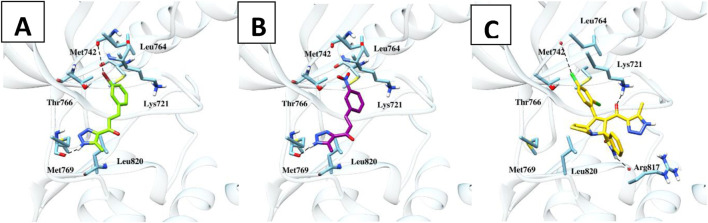
Molecular interactions of the docked compounds **6b**, **6c,** and **9h** (**A, B** and **C**, respectively) inside the EGFR protein using AutoDock software with Chimera for visualization.

Molecular docking studies were conducted on compounds **6b** and **6c** to elucidate their potential binding mechanisms with the EGFR protein. Before docking, redocking was performed to validate the docking protocol. The re-docking results with an rmsd value less than 2 Å confirmed the suitability of autodock as an appropriate software for further docking studies. As illustrated in (Figure 6), compounds **6b** and **6c** retained the binding mode of the co-crystallized ligand and docked within the EGFR binding site, exhibiting binding energies of −18.68 and −18.98 kcal/mol, respectively. Additionally, these compounds engaged in key binding interactions involving Met769 as the pivotal amino acid. Likewise, compound **9h**, identified as a spirooxindole-Triazole derivative, was effectively docked within the EGFR binding site, exhibiting a binding energy of −11.28 kcal/mol. Compound **9h** established two hydrogen bond interactions with Lys721 and Arg817 at distances of 2.3 and 2.7 Å, respectively. Besides hydrogen bond and hydrophobic interactions, compounds **6b** and **9h** were stabilized by halogen bond interactions with the oxygen of Met742. Details of these interactions are provided in Table 5. The above docking study was consistent with experimentally observed cell viability and EGFR-kinase assessment data. The outcomes of molecular docking, along with evidence from the enzyme assay, indicate that compounds **6b, 6c**, and **9h** are potential inhibitors of EGFR.

**TABLE 5:** Predicted docking scores and detailed interactions of target compounds.

Compound	Docking score (kal mol^−1^	Interacting residues		
		H-bond	Hydrophobic	Halogen
Sorafenib	−9.5	Met769	Lys721, Leu764, Thr766, Leu820	Trp-817
6b	−18.68	Met769	Lys721, Leu764, Thr766, Leu820	Met742
6c	−18.98	Met769	Lys721, Leu764, Thr766, Leu820	
9h	−11.28	Lys721, Arg817	Lys721, Leu764, Thr766, Leu820	Met742

##### 3.3.2.1 ADME properties

SwissADME web server was used to find the chemicals that met the requirements of Veber, Egan, and Lipinski rule. For compound **6b, 6c** and **9h**, following parameters were calculated: solubility, synthetic accessibility (SA), hydrogen bond donors (HBD), hydrogen bond acceptors (HBA), number of rotational bonds (n-ROTB), and %F bioavailability score (Supplementary Table S1 in Supplementary Material). All three molecules were examined complies with Veber, Egan, and Lipinski’s guidelines. The synthetic accessibility of the molecules was found to be less than 10, which indicates that these molecules are easy to synthesize. Additionally, each molecule has a high bioavailability (%F > 50%) and zero pan-assay interference warnings (PAINS), meaning that it could be used as a reference compound.

The findings show that these compounds can readily pass through the digestive system barrier due to their high intestinal absorption rate of more than 70%.

##### 3.3.2.2 Molecular dynamic simulation

**FIGURE 7:**
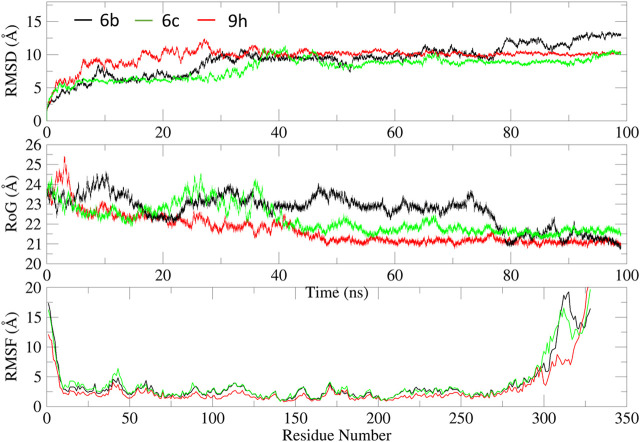
MD analysis of compounds **6b** (black), **6c** (green), and **9h** (red) with EGFR protein: **(A)** RMSD, **(B)** RoG, and **(C)** RMSF with respect to time.

We performed 100 ns molecular dynamics (MD) simulations for the three most active compounds to study their binding strength to the EGFR protein. The trajectory data were used to extract the root-mean-square deviation (RMSD, Figure 7A), root-mean-square fluctuation (RMSF, Figure 7C), and radius of gyration (RoG, Figure 7B).

As evident from Figure 7, all systems displayed variable deviations, primarily due to significant fluctuations in the loop regions (Gly672-Lys684, Thr830-Pro853, and Tyr954-Gly998), as reflected in the RMSF values (Figure 7C). Additionally, the terminal regions exhibited very large fluctuations, reaching up to 12 Å. This observation is further supported by the analysis of RoG, which suggests varying degrees of compactness throughout the simulation.

## 4 Conclusion

In conclusion, a novel series of spirooxindole derivatives, initially designed through a systematic multistep synthetic approach to incorporate a triazolyl-s-triazine framework, unexpectedly revealed an alternative synthetic route leading to the formation of spirooxindole derivatives with a triazole motif. A detailed exploration of the reaction mechanism of the 32CA reaction of AY **(10m)** with ethylene **(6m)** using MEDT provided valuable insights. The synthesized compounds underwent rigorous evaluation for antiproliferative efficacy against MDA-MB-231 and HepG2 cell lines, with chalcones (**6c** and **6d**) demonstrating notable activity. Moreover, spirooxindoles (**9b, 9c, 9h,** and **9i**), especially those with a 2,4-dichlorophenyl moiety, exhibited remarkable potency, surpassing the activity of Sorafenib, a positive control. These findings underscore the potential of the synthesized spirooxindole derivatives as promising candidates for further exploration in anticancer drug development.

## Data Availability

The original contributions presented in the study are included in the article/Supplementary Material, further inquiries can be directed to the corresponding authors.
