# Supplementary material for: Novel spirooxindole-triazole derivatives: unveiling [3+2] cycloaddition reactivity through molecular electron density theory and investigating their potential cytotoxicity against HepG2 and MDA-MB-231 cell lines
Source: Front Chem. 2024 Sep 30;12:1460384. doi: 10.3389/fchem.2024.1460384 (PMC11471631; doi:10.3389/fchem.2024.1460384)
Supplement: Supplementary file 1 [file DataSheet1.pdf]

SUPPORTING INFORMATION

Novel Spirooxindole-Triazole Derivatives: Unveiling [3+2] Cycloaddition Reactivity through Molecular Electron Density Theory and their potential cytotoxicity against HepG2 and MDA-MB-231 cell lines

Ihab Shawish [1, *], Samha Al Ayoubi [1], Ayman El-Faham [2,3,*], Ali Aldalbahi [4], Fardous F. El-Senduny [5,6], Farid A. Badria [7], Mar Ríos-Gutiérrez [8], Hassan H. Hammud [9], Sajda Ashraf [10], Zaheer Ul-Haq [10], Assem Barakat [4]

[1] Department of Math and Sciences, College of Humanities and Sciences, Prince Sultan University, P.O. Box 66833, Riyadh 11586, Saudi Arabia, ishawish@psu.edu.sa (I.S.); sayoubi@psu.edu.sa (S.A.).

[2] Department of Clinical Sciences, College of Medicine, Dar Al Uloom University, Al Falah, Riyadh 13314, Saudi Arabia, ayman.a@dar.edu.sa (A. E-F)

[3] Chemistry Department, Faculty of Science, Alexandria University, P.O. Box 426, Ibrahimia, 12321 Alexandria, Egypt, ayman.elfaham@alexu.edu.eg; aymanel_faham@hotmail.com (A.E-F.).

[4] Department of Chemistry, College of Science, King Saud University, P.O. Box 2455, Riyadh 11451, Saudi Arabia, aaldalbahi@ksu.edu.sa (A.D.).

[5] Department of Pathology & Laboratory Medicine, Sylvester Comprehensive Cancer Center, Miller School of Medicine, Miami, FL, 33136, United States. Fxe123@miami.edu (F.F.E.-S.).

[6] Department of Chemistry, Faculty of Science, Mansoura University, Mansoura, Egypt.

[7] Department of Pharmacognosy, Faculty of Pharmacy, Mansoura University, Mansoura 35516 Egypt.

[8] Department of Organic Chemistry, University of Valencia, Dr. Moliner 50, 46100 Burjassot, Valencia, Spain.

[9] Department of Chemistry, College of Science, King Faisal University, P.O. Box 400, Al-Ahsa 31982, Saudi Arabia.

[10] Dr. Panjwani Center for Molecular medicine and Drug Research, International Center for Chemical and Biological Sciences, University of Karachi, Karachi-75270, Pakistan. sajda.ashraf@yahoo.com (S.A.); zaheer_qasmi@hotmail.com (Z.U.-H.).

* Correspondence: ishawish@psu.edu.sa (I.S.); ambarakat@ksu.edu.sa (A.B.).

Tables:

Table S1. Selected copy of the (^{1}HNMR and ^{13}CNMR) spectrum of the synthesized compounds.

**Figure S1**	^{1}H and ^{13}C of **4a**
**Figure S2**	^{1}H and ^{13}C of **4b**
**Figure S3**	^{1}H and ^{13}C of **6a**
**Figure S4**	^{1}H and ^{13}C of **6b**
**Figure S5**	^{1}H and ^{13}C of **6c**
**Figure S6**	^{1}H and ^{13}C of **6d**
**Figure S7**	^{1}H and ^{13}C of **9a**
**Figure S8**	^{1}H and ^{13}C of **9b**
**Figure S9**	^{1}H and ^{13}C of **9c**
**Figure S10**	^{1}H and ^{13}C of **9d**
**Figure S11**	^{1}H and ^{13}C of **9e**
**Figure S12**	^{1}H and ^{13}C of **9f**
**Figure S13**	^{1}H and ^{13}C of **9g**
**Figure S14**	^{1}H and ^{13}C of **9h**
**Figure S15**	^{1}H and ^{13}C of **9i**

Table S2. *ω*B97XD/6-311G(d,p) total electronic energies (E, in a.u.), enthalpies (H, in a.u.), entropies (S, in cal·mol^{-1}K^{-1}) and Gibbs free energies (G, in a.u.), computed at 65 ºC in methanol, of the stationary points involved in the 32CA reaction of AY **(10m)** with ethylene **(6m)**.

	H	S	G
AY **10m**	-1007.835756	115.7	-1007.898041
**6m**	-703.049635	124.3	-703.116535
**MC-on**	-1710.916915	196.5	-1711.022683
**TS-on**	-1710.904296	185.8	-1711.004317
**TS-ox**	-1710.894430	187.3	-1710.995231
**TS-mn**	-1710.897732	190.4	-1711.000224
**TS-mx**	-1710.894040	191.4	-1710.997058
**9m**	-1710.955591	187.4	-1711.056471
**11m**	-1710.963496	190.7	-1711.066158
**12m**	-1710.960951	189.0	-1711.062707
**13 m**	-1710.966520	190.8	-1711.069210

Table S3: ADME properties of the most active compound of the series**.**

Molecule	6b	6c	9h
MW	292.13	258.23	500.4
Fraction Csp3	0.08	0.08	0.3
#Rotatable bonds	3	4	3
#H-bond acceptors	3	5	5
#H-bond donors	1	1	2
MR	68.86	69.98	134.96
TPSA	58.64	104.46	116.28
iLOGP	2.09	0.96	2.32
XLOGP3	2.86	2	3.96
WLOGP	2.66	1.81	3.18
MLOGP	1.93	1.11	2.86
Silicos-IT Log P	3.52	0.69	4.18
Consensus Log P	2.61	1.31	3.3
ESOL Log S	-3.73	-2.87	-5.62
GI absorption	High	High	High
log Kp (cm/s)	-6.05	-6.46	-6.54
Lipinski #violations	0	0	1
Ghose #violations	0	0	2
Veber #violations	0	0	0
Egan #violations	0	0	0
Muegge #violations	0	0	0
Bioavailability Score	0.55	0.55	0.55
PAINS #alerts	0	0	0
Brenk #alerts	1	3	0
Leadlikeness #violations	0	0	2
Synthetic Accessibility	2.75	2.76	5.23

Molecular docking

"Utilizing Maestro, protein and compound structures were created and optimized. Binding sites inside proteins were then identified using the grid-box dimensions surrounding the co-crystallized ligands. The investigated compounds were docked against the protein structures of EGFR (PDB = 1M17) using AutoDock Vina software following routine work Maestro was

utilized to enhance protein and ligand structures and to energetically favor them. In terms of binding energy and ligand-receptor interactions, binding activities evaluated the results of molecular docking. Chimera was then used to complete the visualization.

Biology

Cell lines and reagents

HepG2 and MDA-MB-231 lines were purchased from ATCC. Cells were grown in DMEM (Biowest, France) supplemented with bovine serum albumin (10%, Life Science Group L, UK, Cat No: S-001B-BR) and with 100 IU/mL penicillin/ streptomycin (100 µg/mL) (Lonza, 17-602E). The tested compounds were prepared in dimethyl sulfoxide (10 mM stock) (DMSO Cat. No. 20385.02, Serva, Heidelberg, Germany) and stored at –20°C.

The initial screening and cell viability by MTT assay

The cancer cells were seeded in a 96-well plate (100 µL/well). After overnight incubation at 37°C and 5% CO_2, the cells were incubated with 50 µM of each tested compound or DMSO (0.5% V/V). After 48 hours of incubation, MTT (3-(4,5-dimethylthiazoyl)-2,5-diphenyl-tetrazolium bromide (MTT) (5 mg/mL Phosphate Buffered Saline (PBS)) was added, and the plate was incubated for 4 hours. After that, acidified sodium dodecyl sulfate (SDS) solution (10% SDS containing 0.01N HCl in 1x PBS) was used to solubilize formazan crystals. The absorbance was measured after 14 hours of incubation at $\lambda_{570-630}$ nm by a Biotek plate reader (Gen5™).

NMR CHARTS

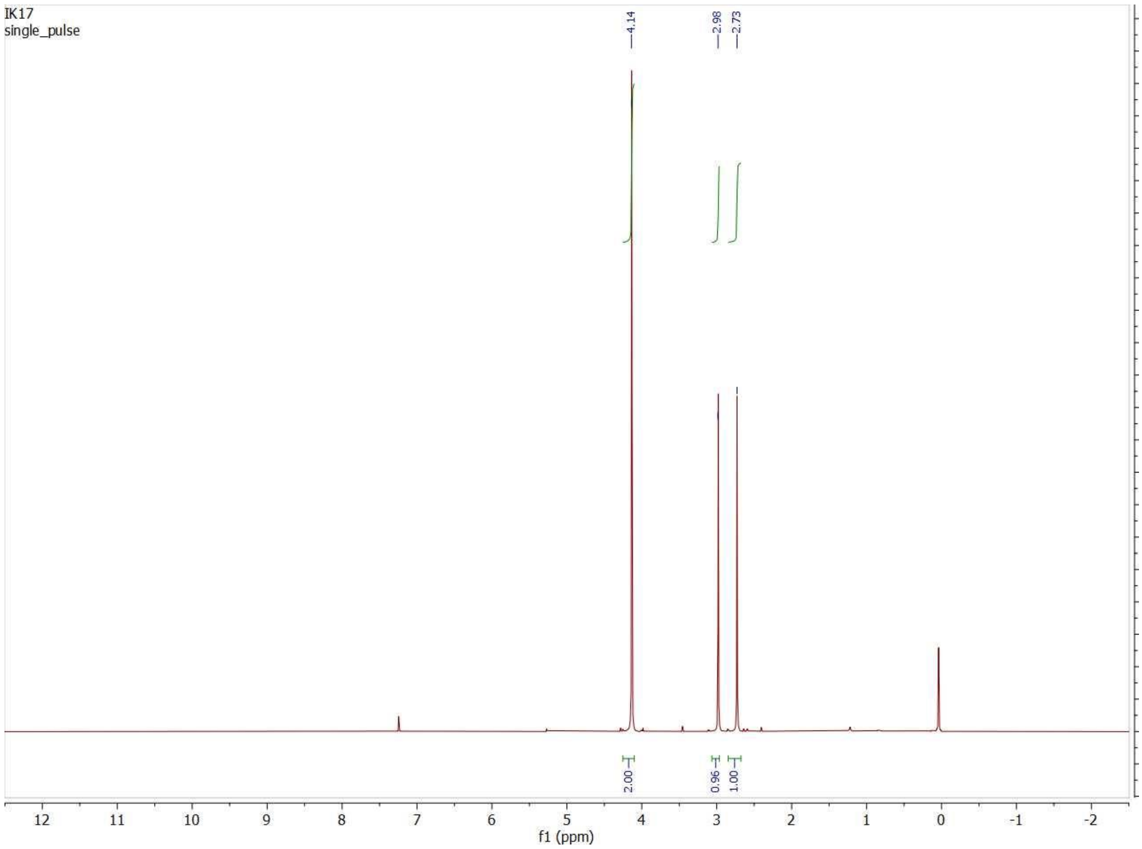

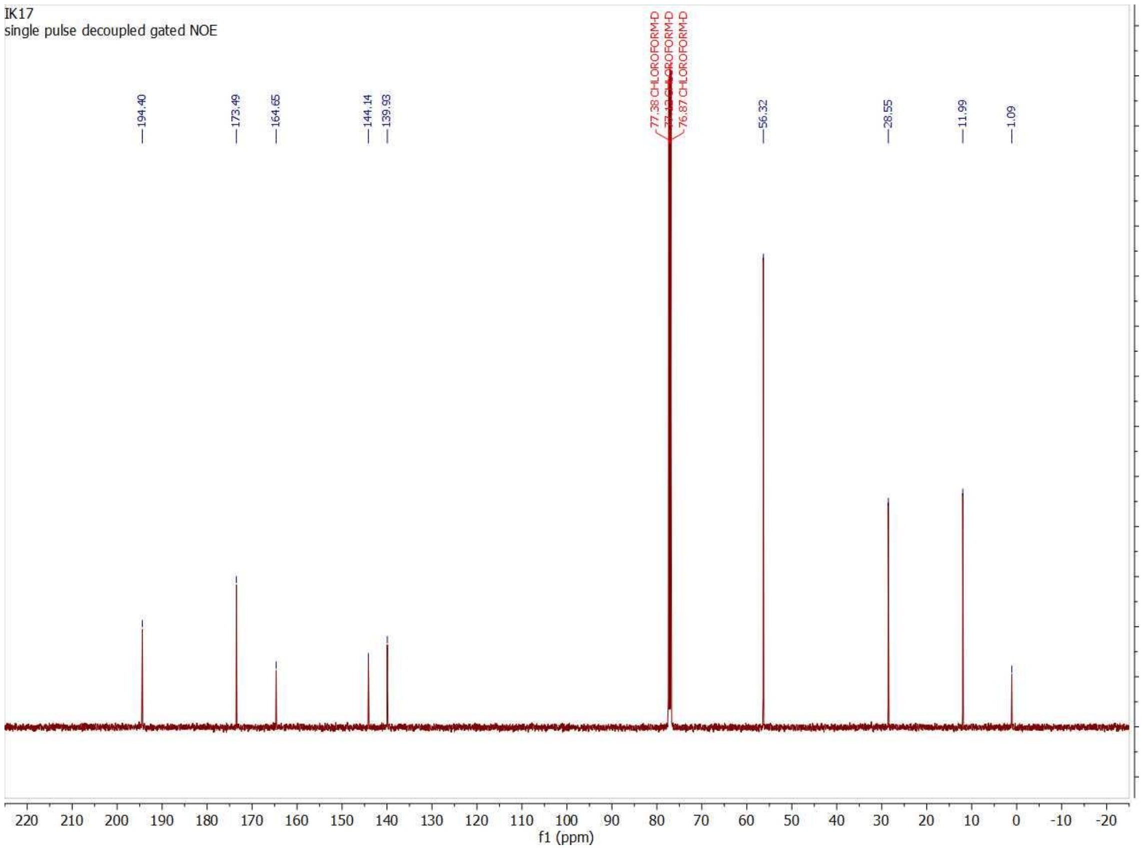

Figure S1. ^{1}H-NMR and ^{13}C-NMR of **4a**

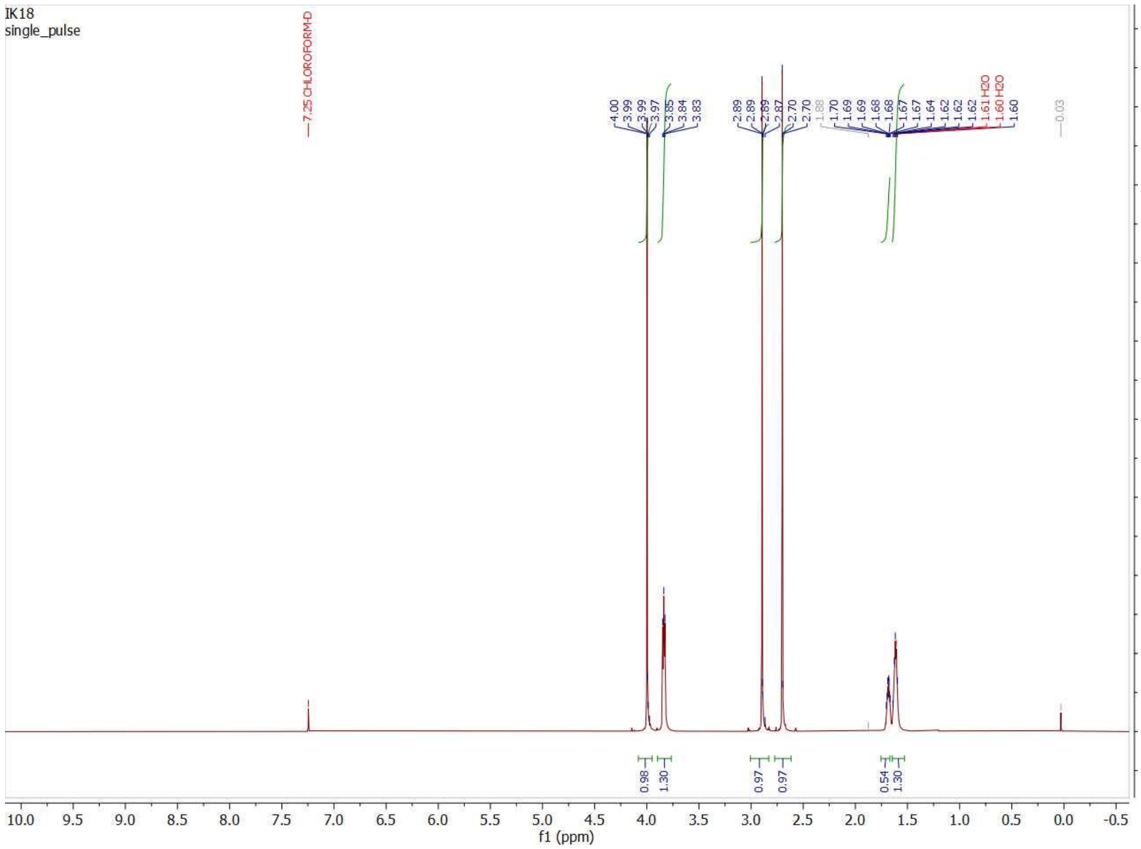

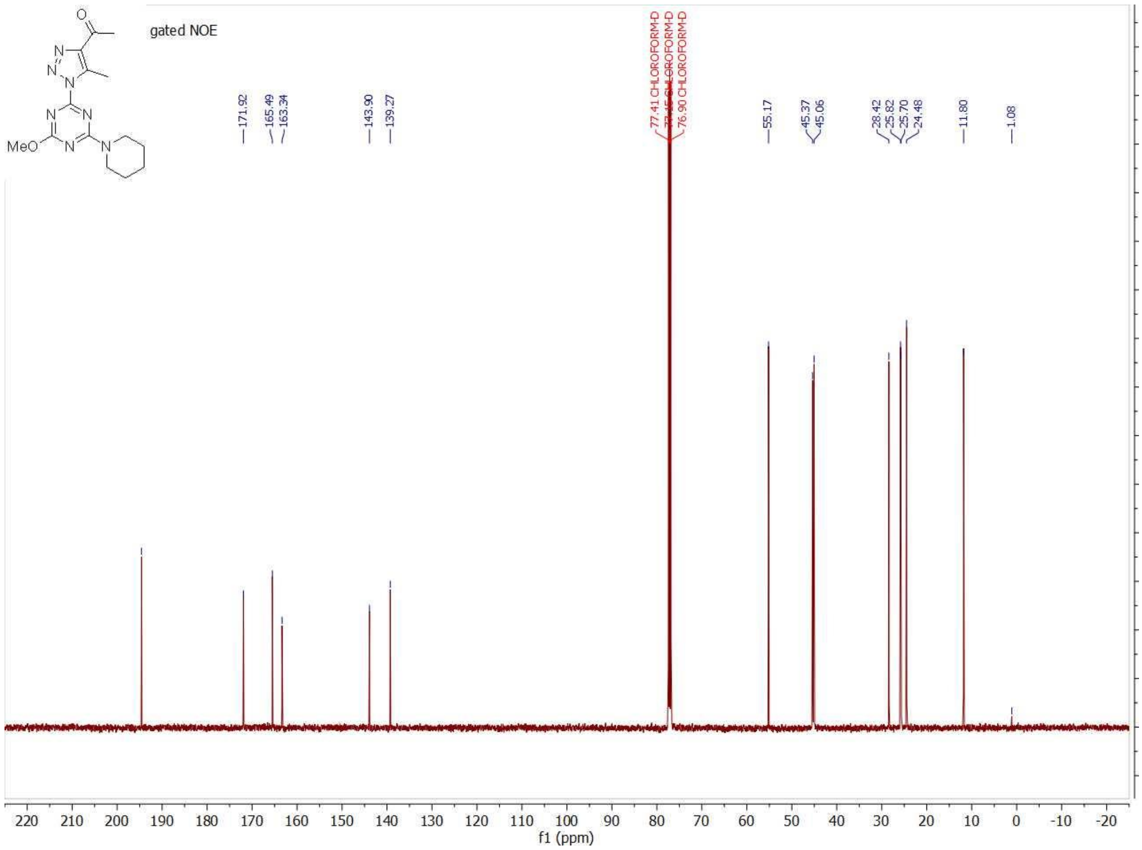

Figure S2. ^{1}H-NMR and ^{13}C-NMR of **4b**

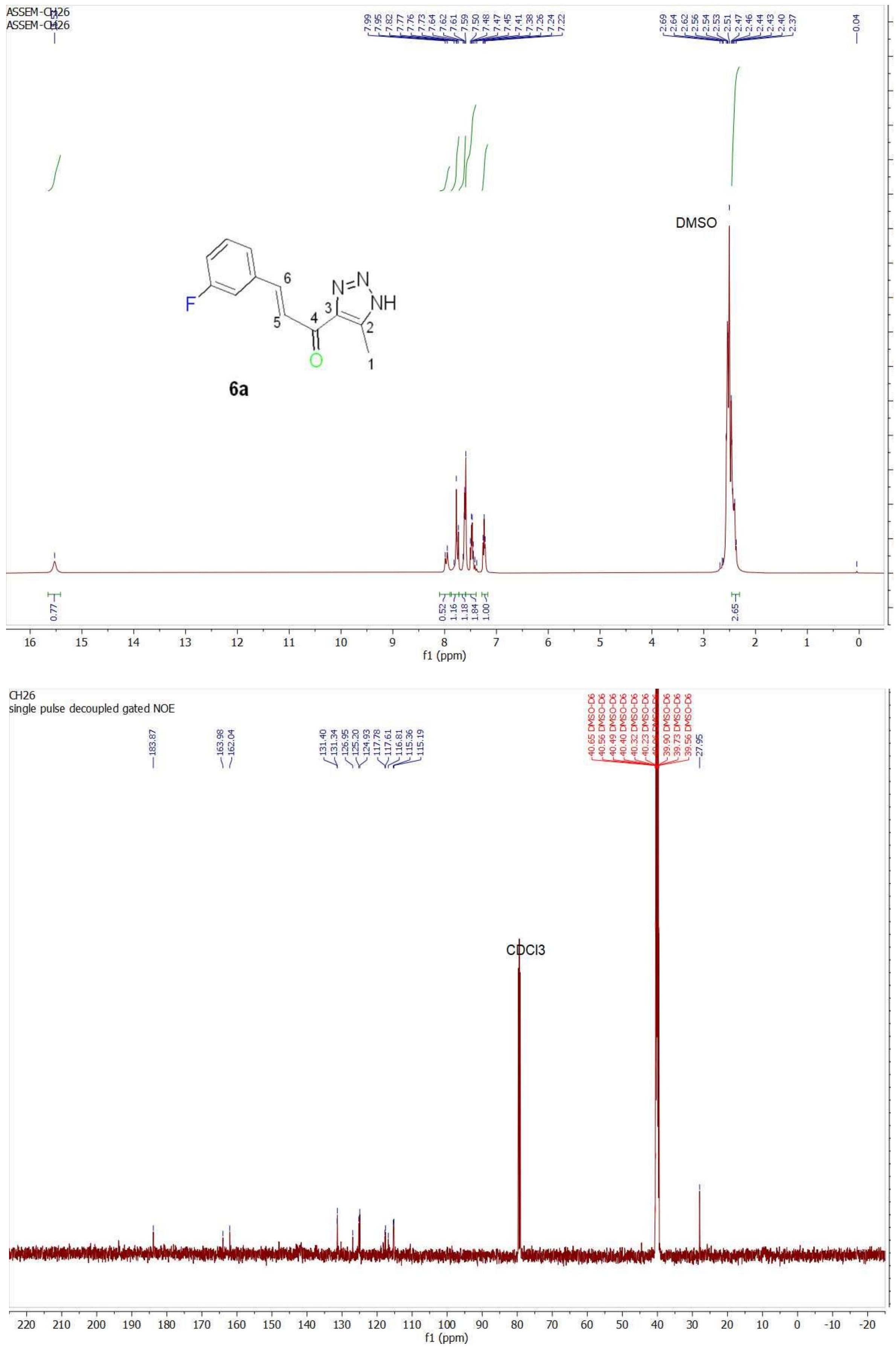

Figure S3. ^{1}H-NMR and ^{13}C-NMR of **6a**

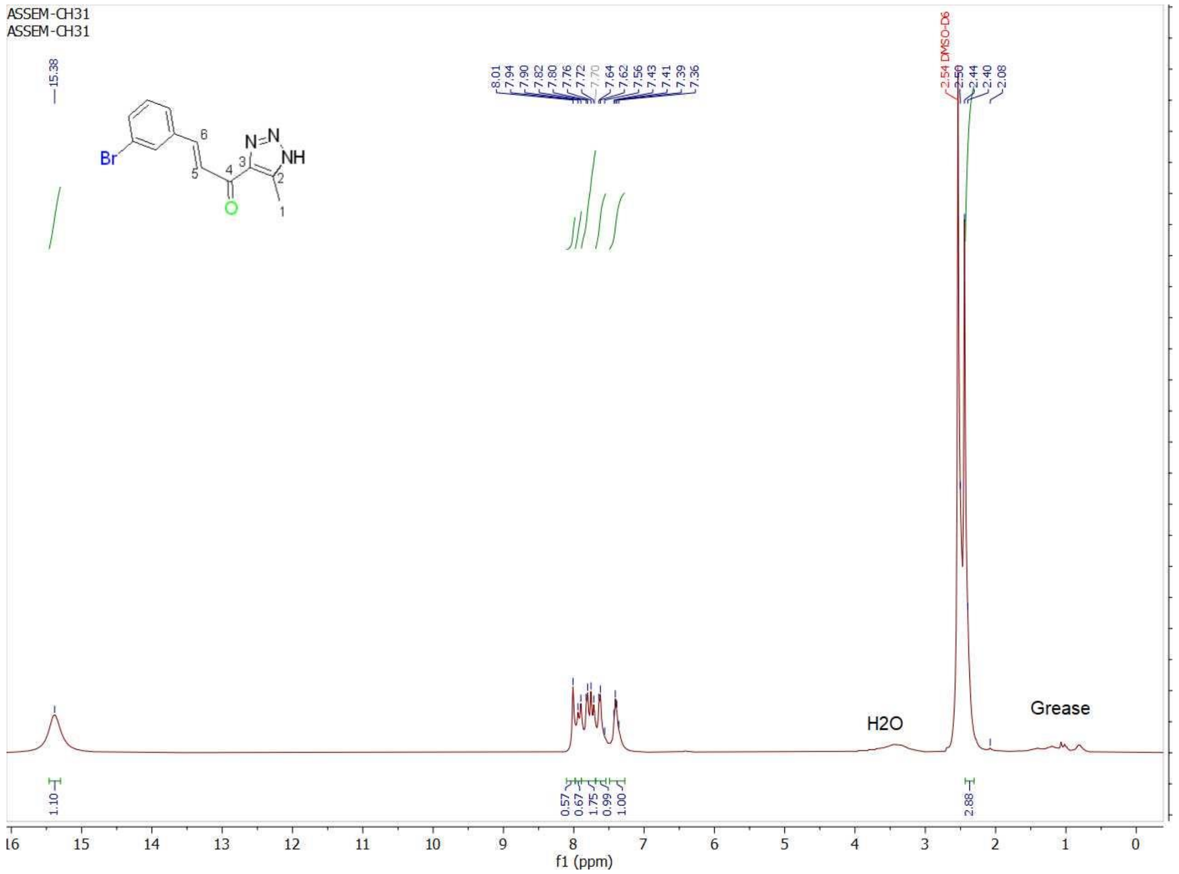

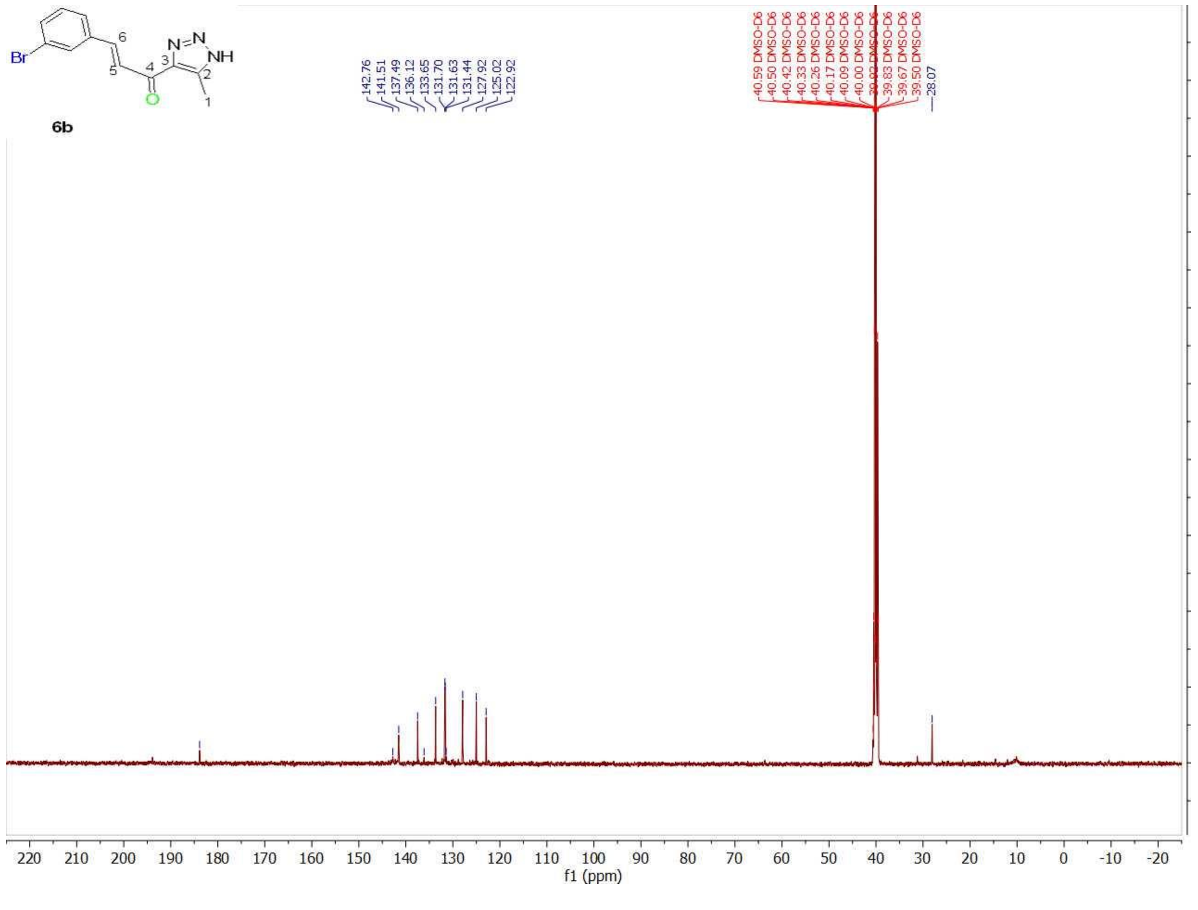

Figure S4: ^{1}H-NMR and ^{13}C-NMR of **6b**

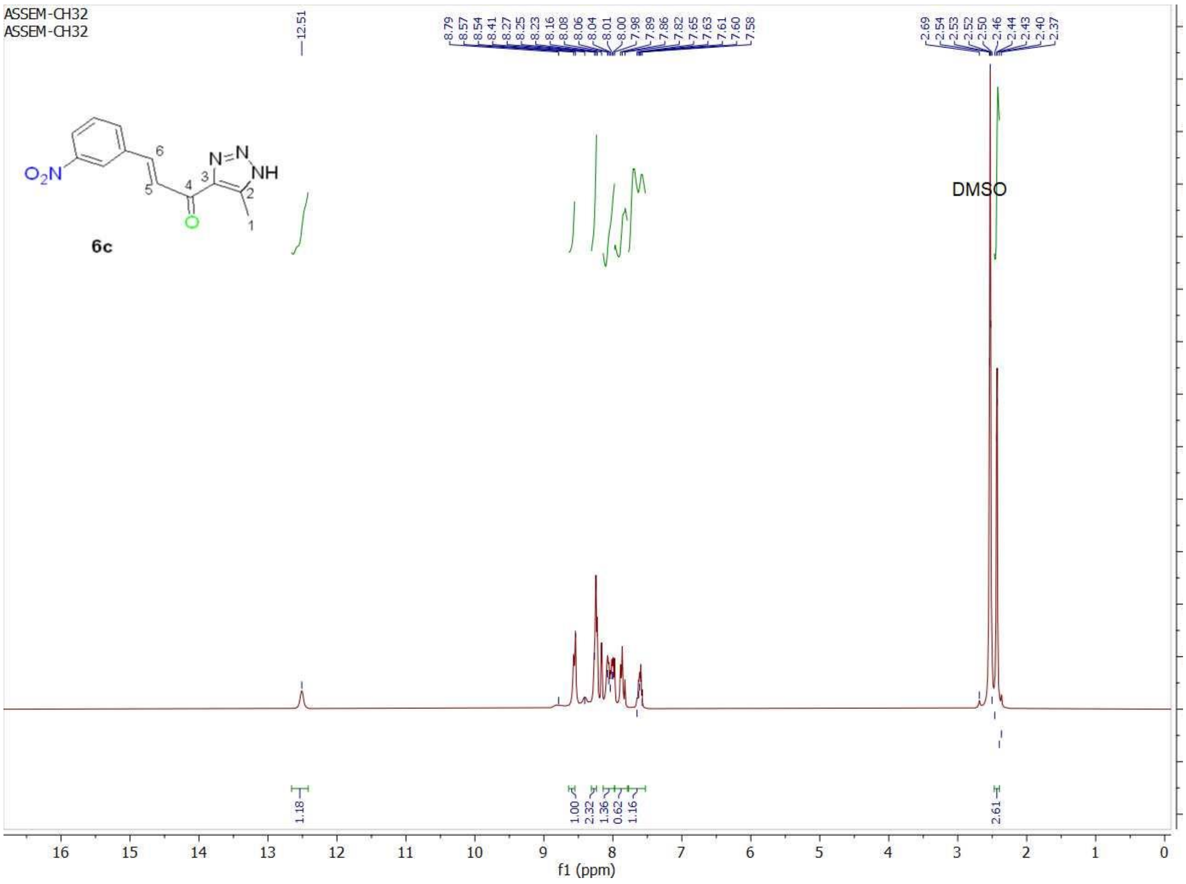

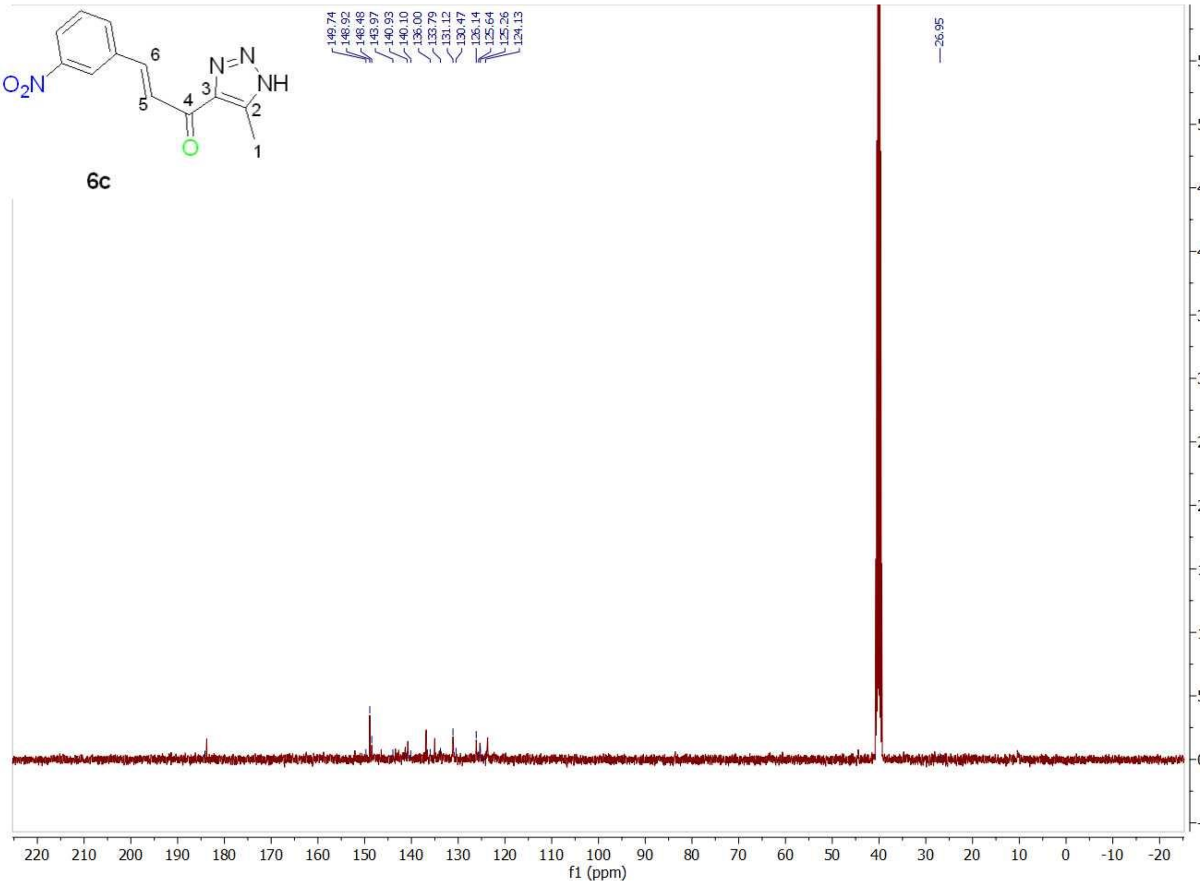

Figure S5. ^{1}H-NMR and ^{13}C-NMR of **6c**

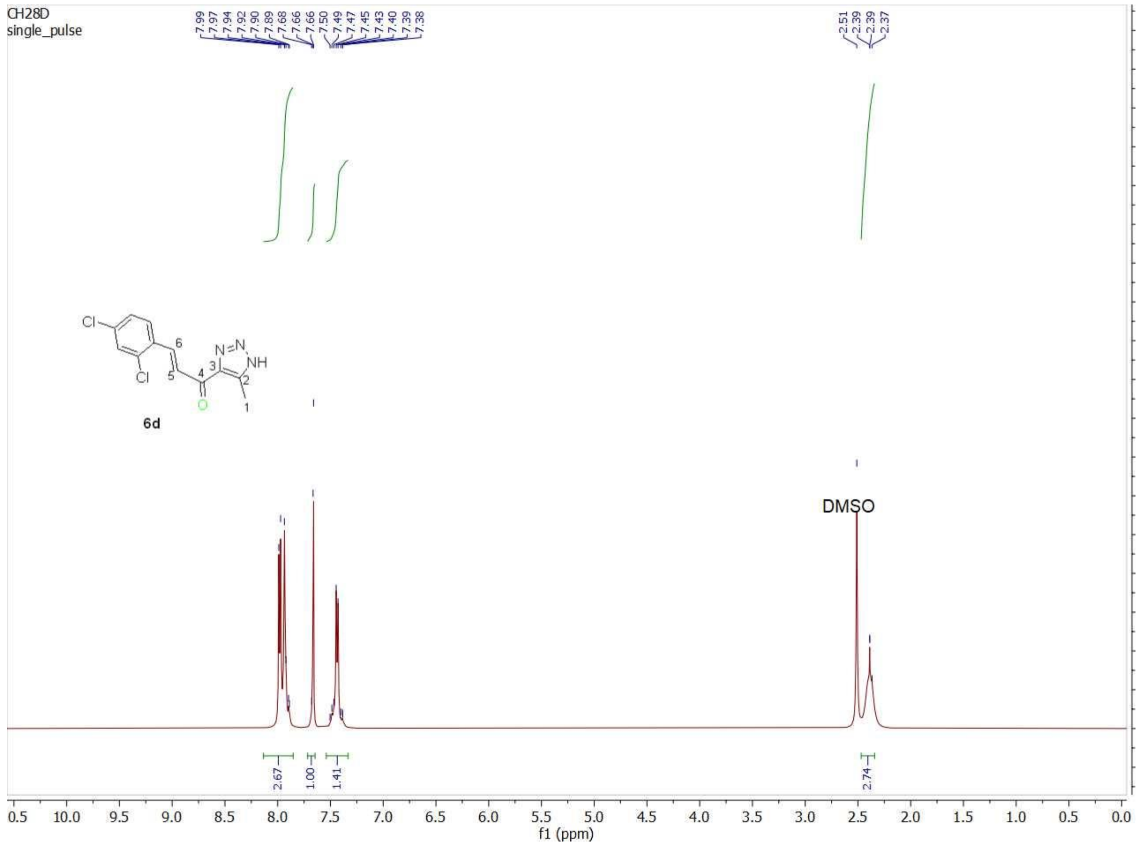

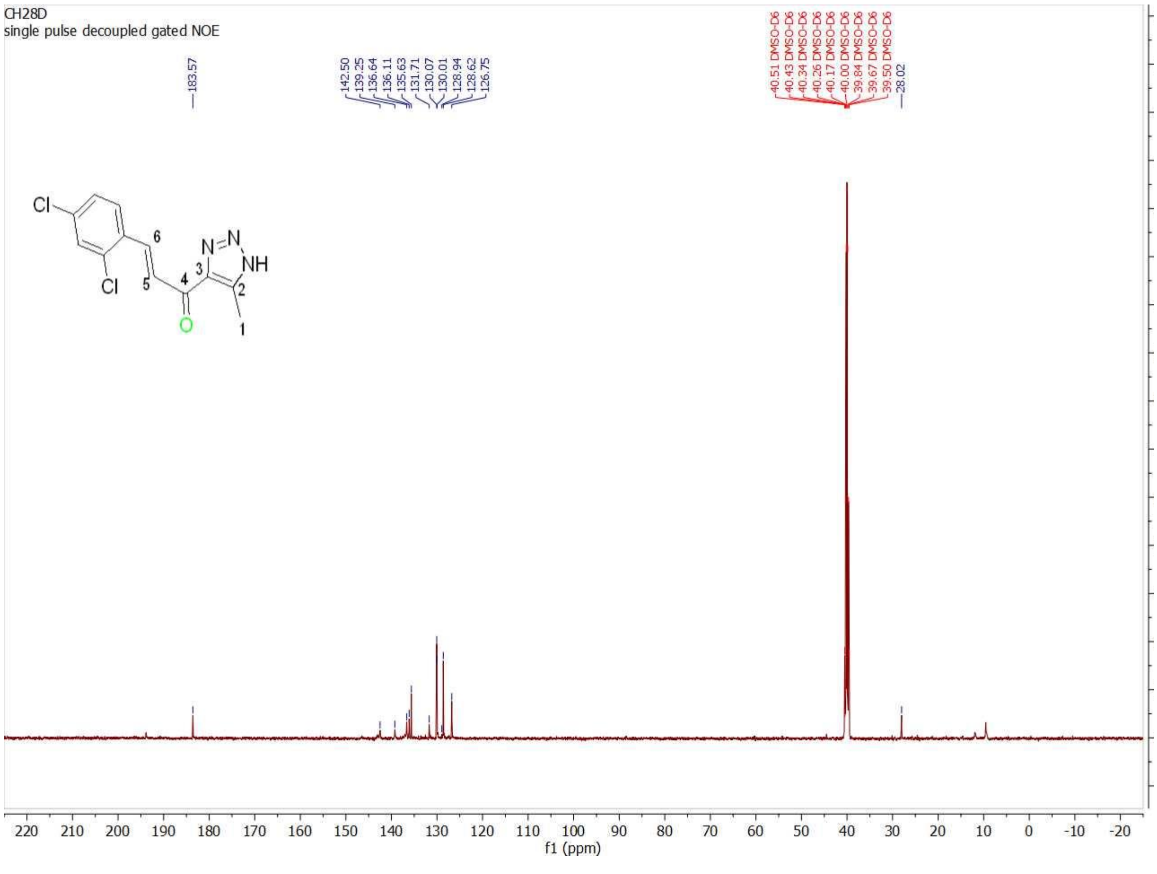

Figure S6: ^{1}H-NMR and ^{13}C-NMR of **6d**

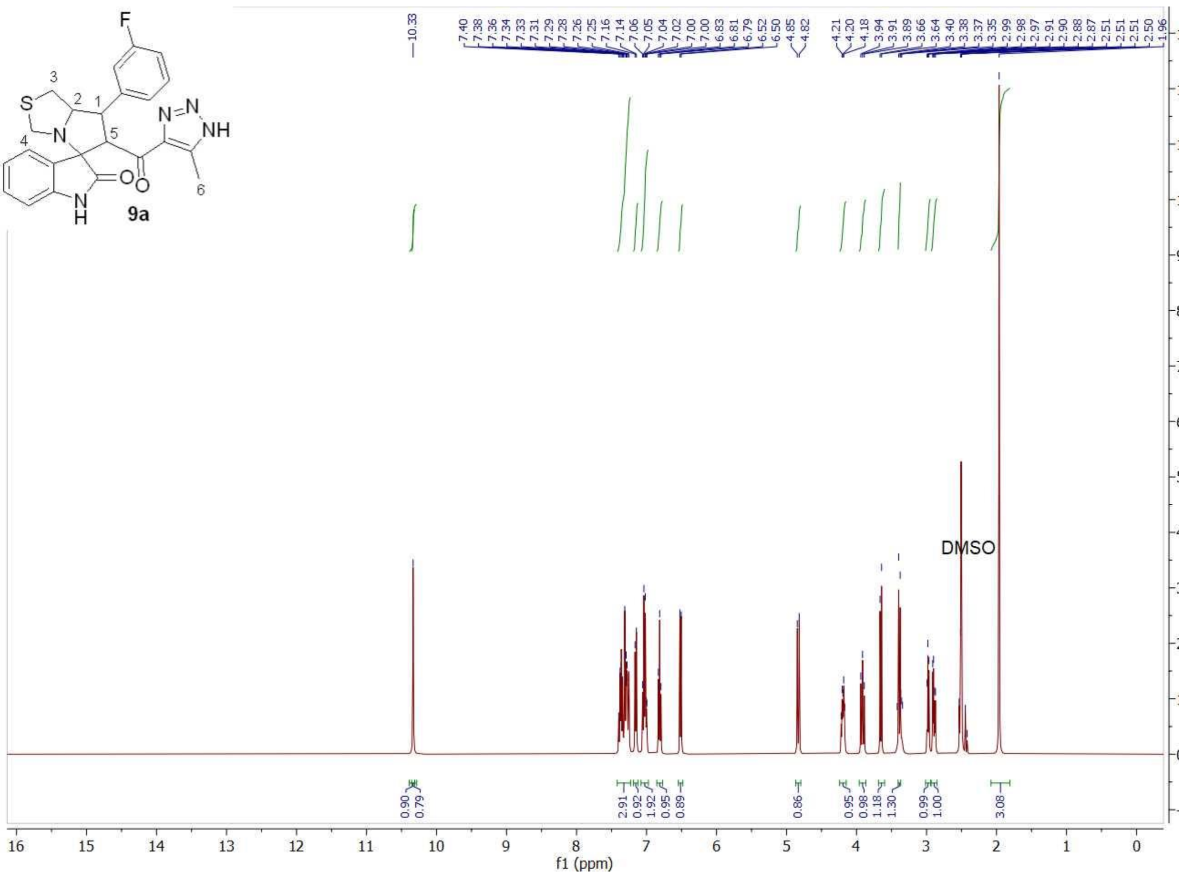

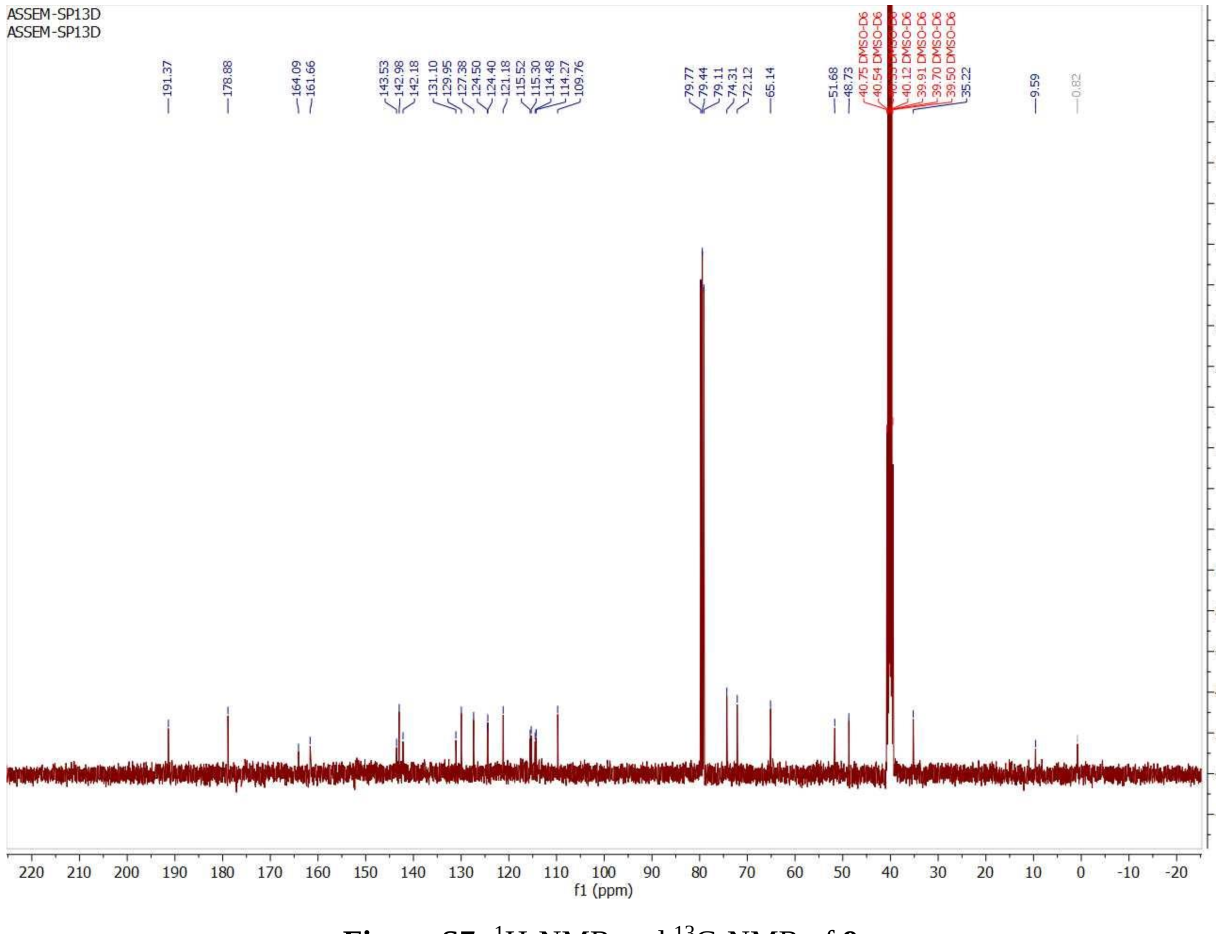

Figure S7: ^{1}H-NMR and ^{13}C-NMR of **9a**

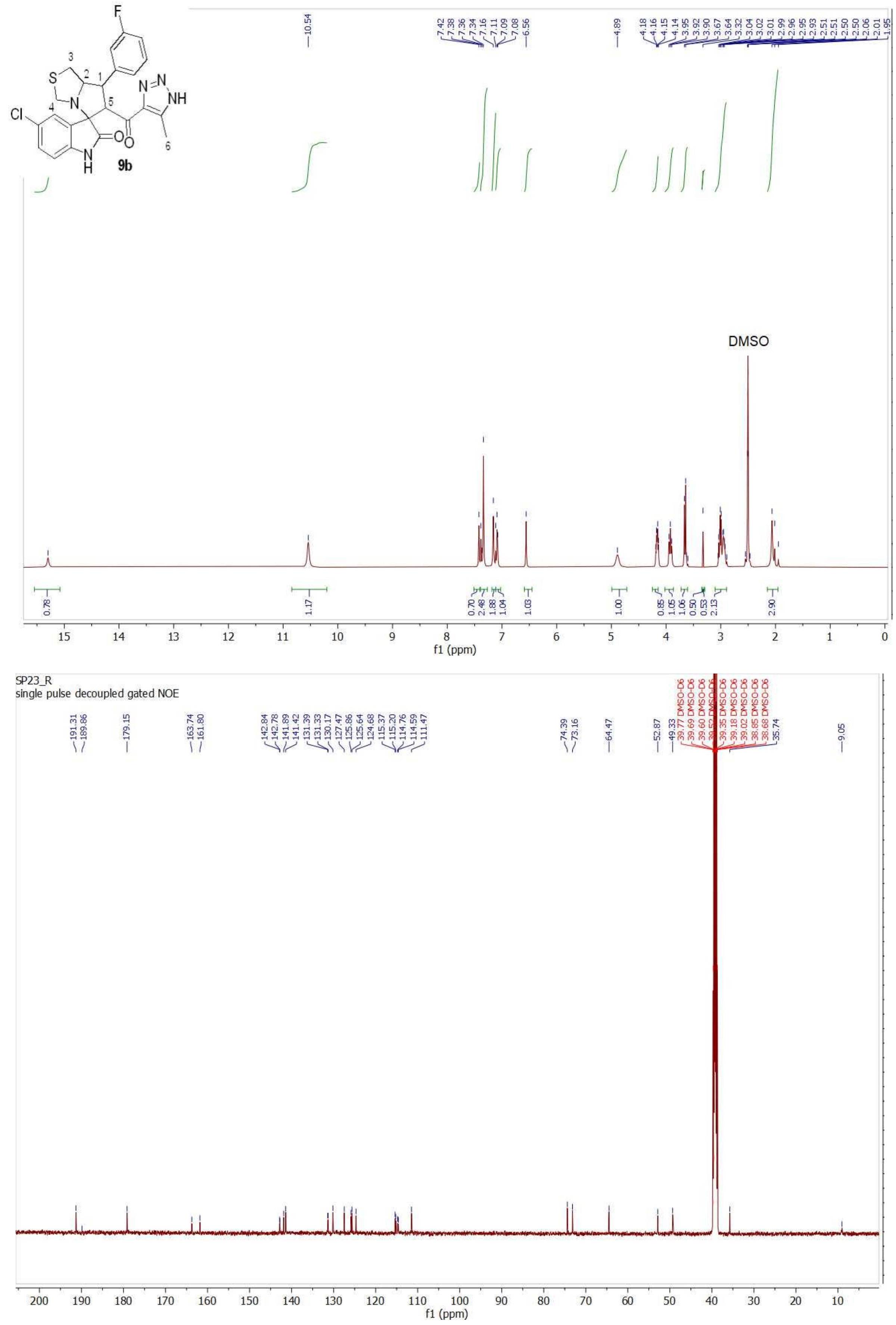

Figure S8: ^{1}H-NMR and ^{13}C-NMR of **9b**

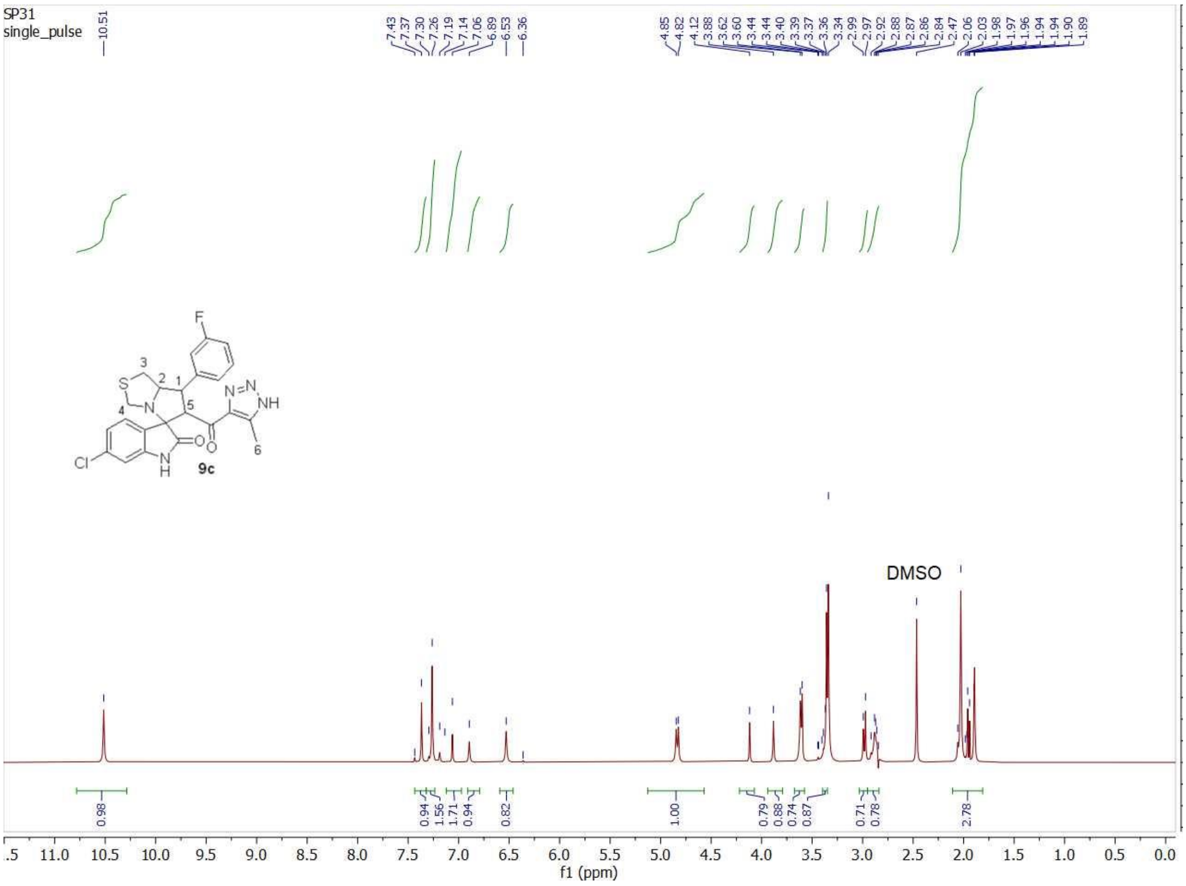

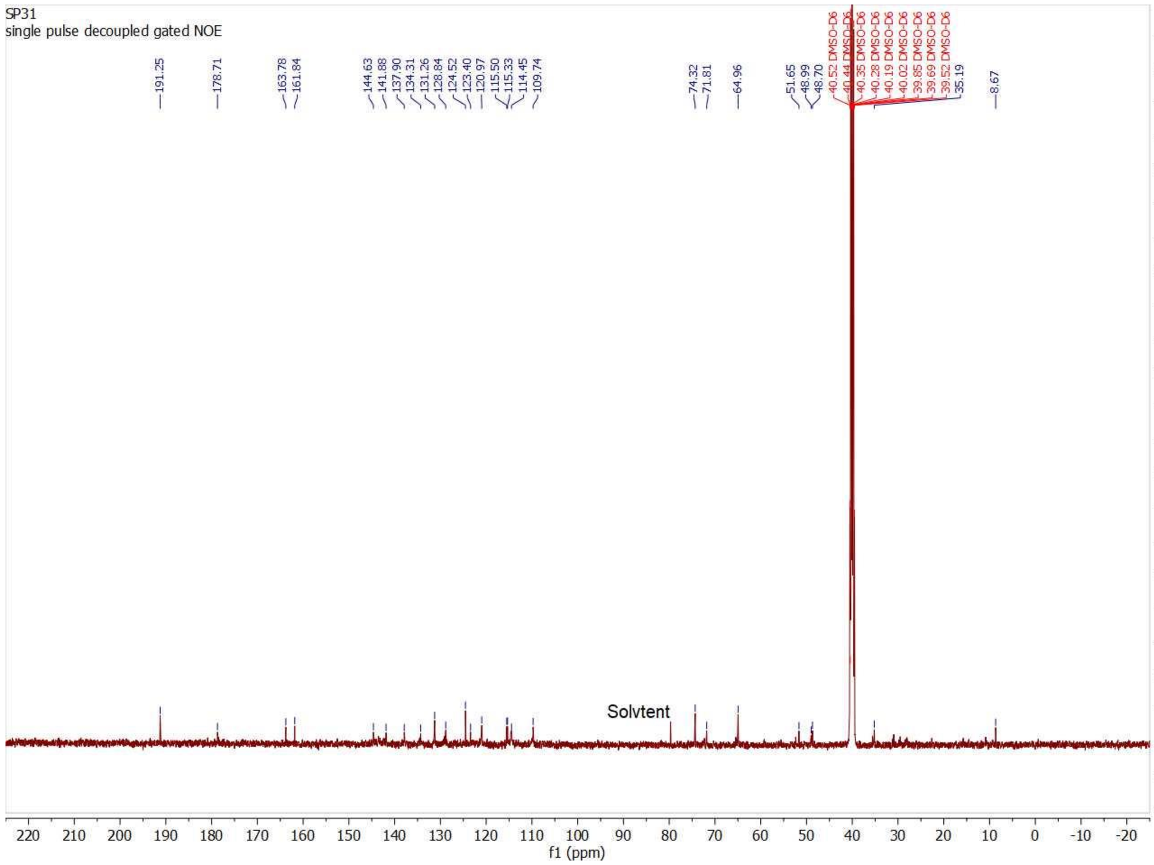

Figure S9: ^{1}H-NMR and ^{13}C-NMR of **9c**

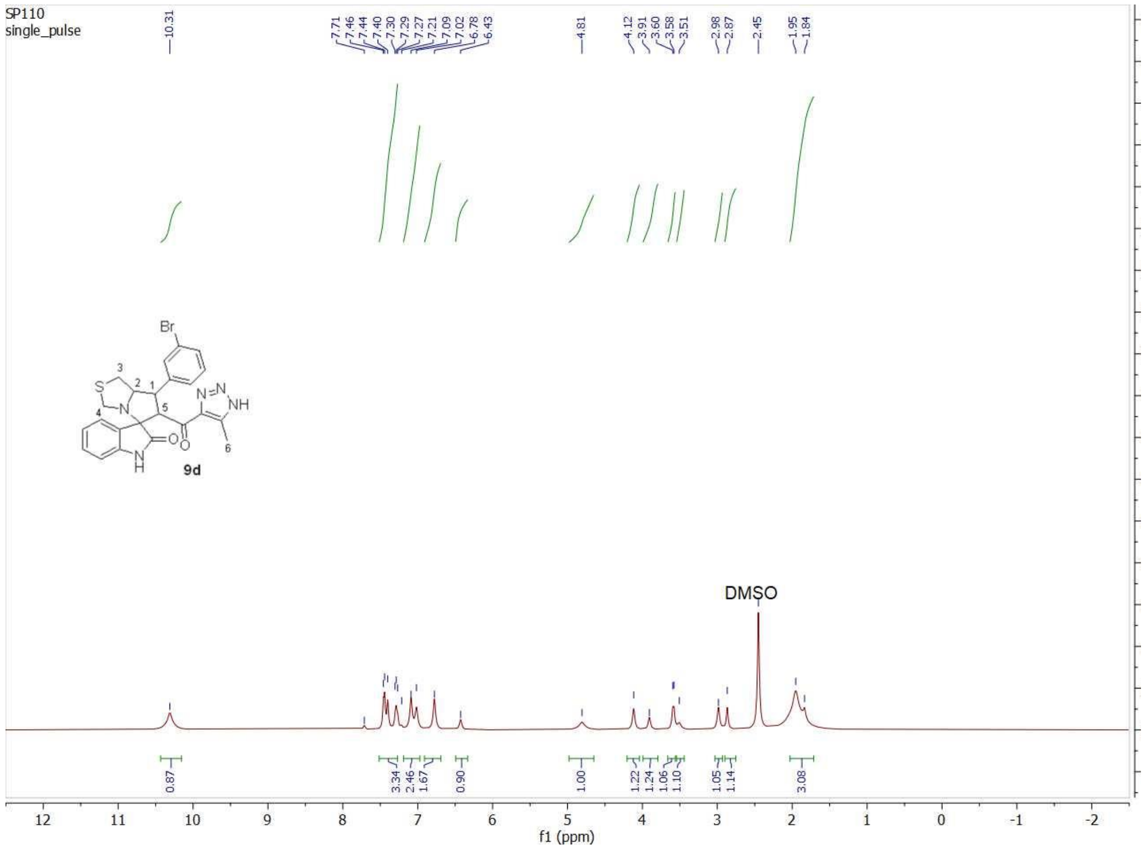

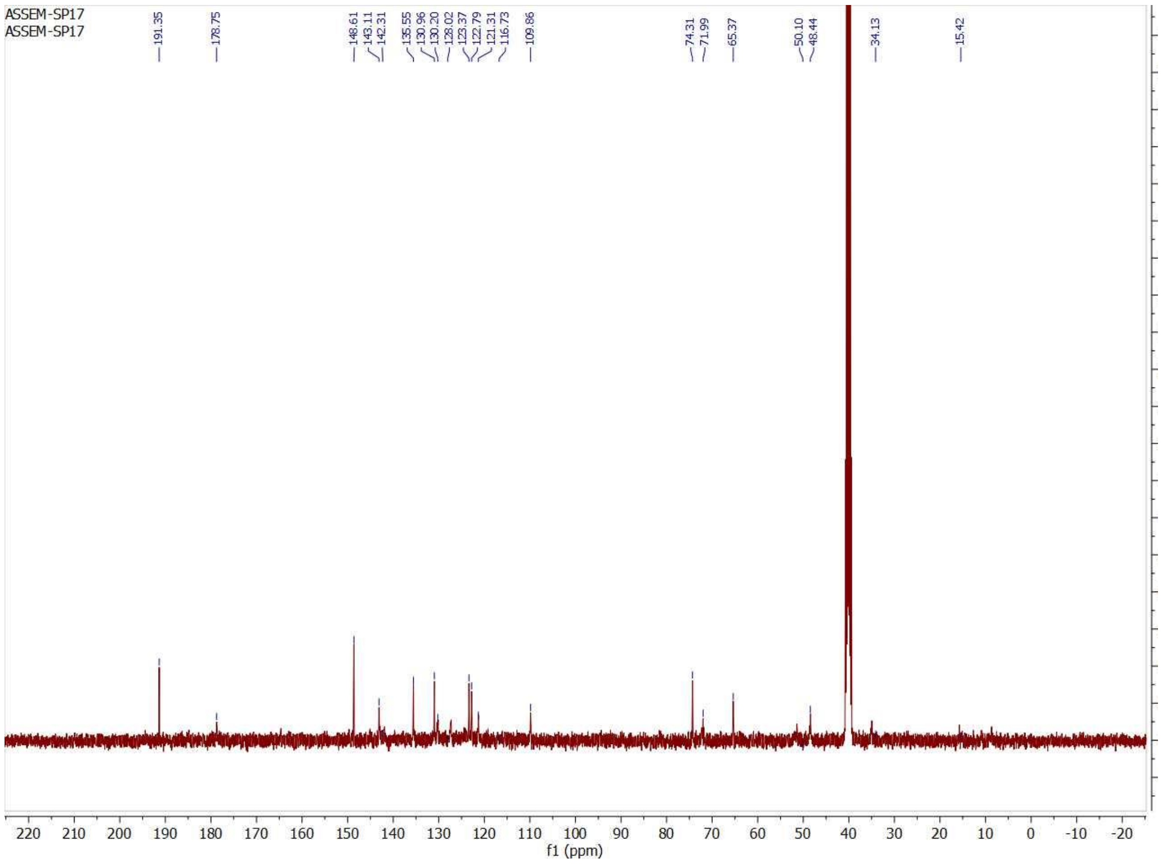

Figure S10: ^{1}H-NMR and ^{13}C-NMR of **9d**

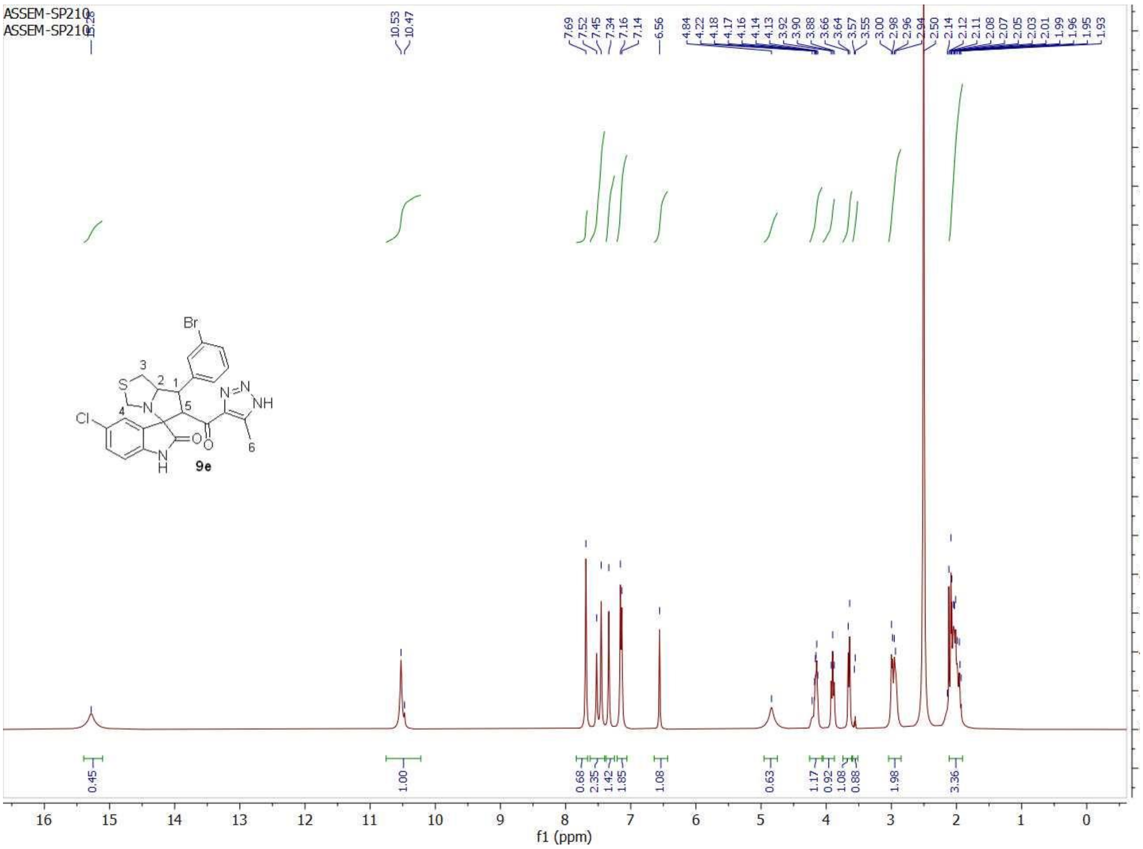

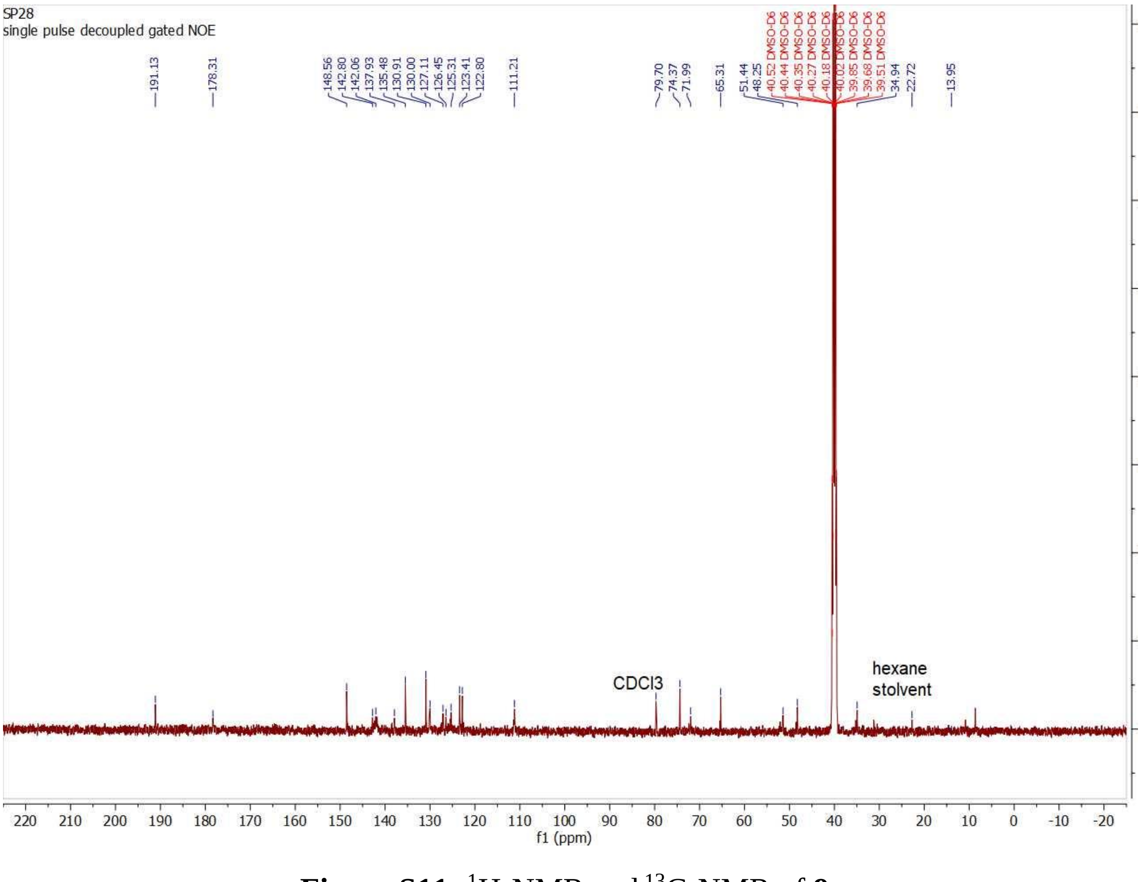

Figure S11: ^{1}H-NMR and ^{13}C-NMR of **9e**

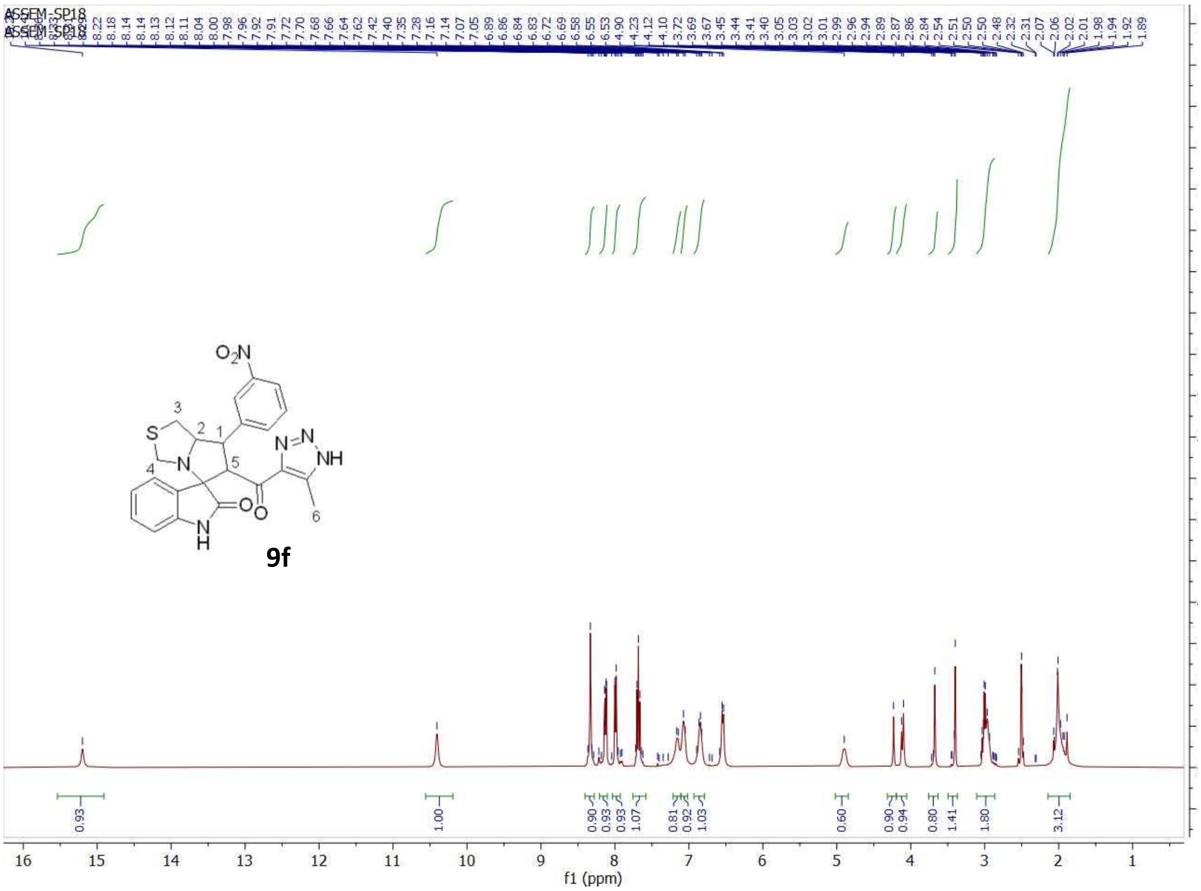

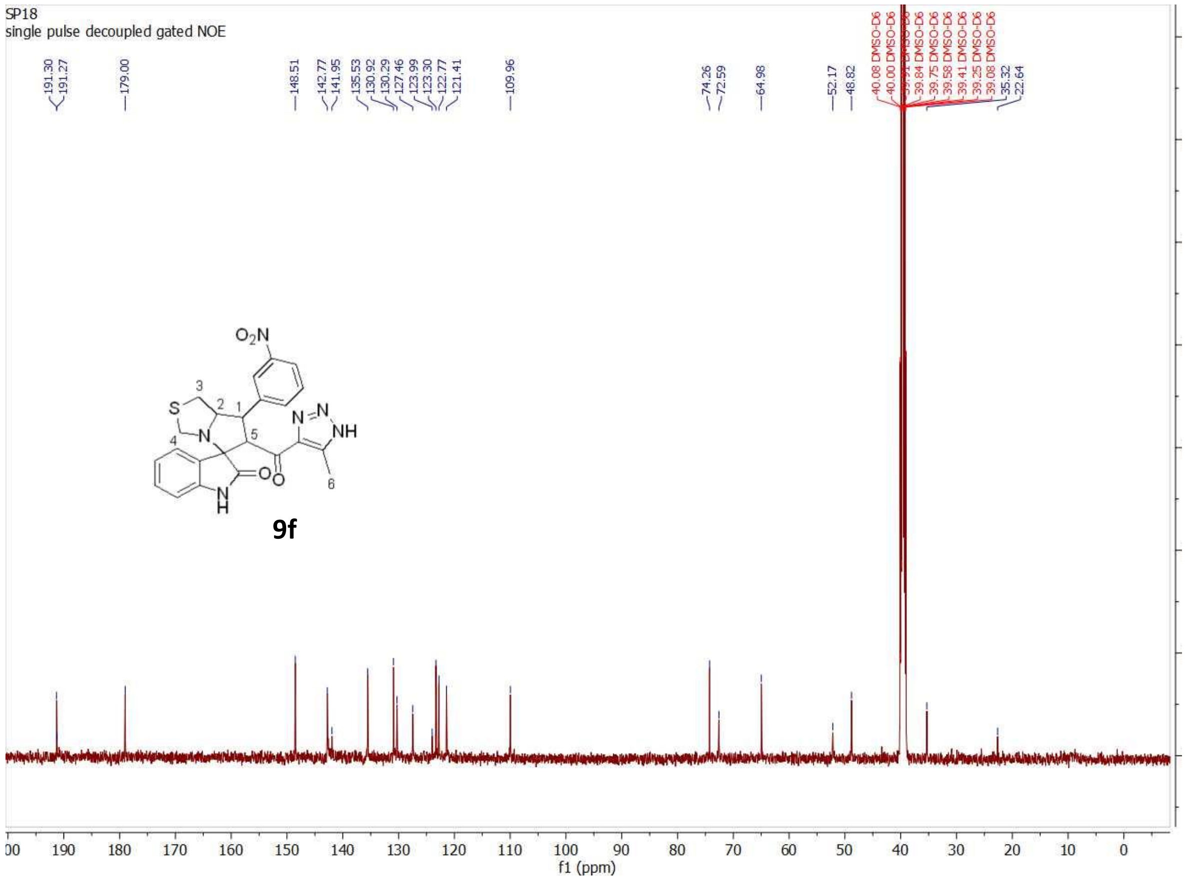

Figure S12: ^{1}H-NMR and ^{13}C-NMR of **9f**

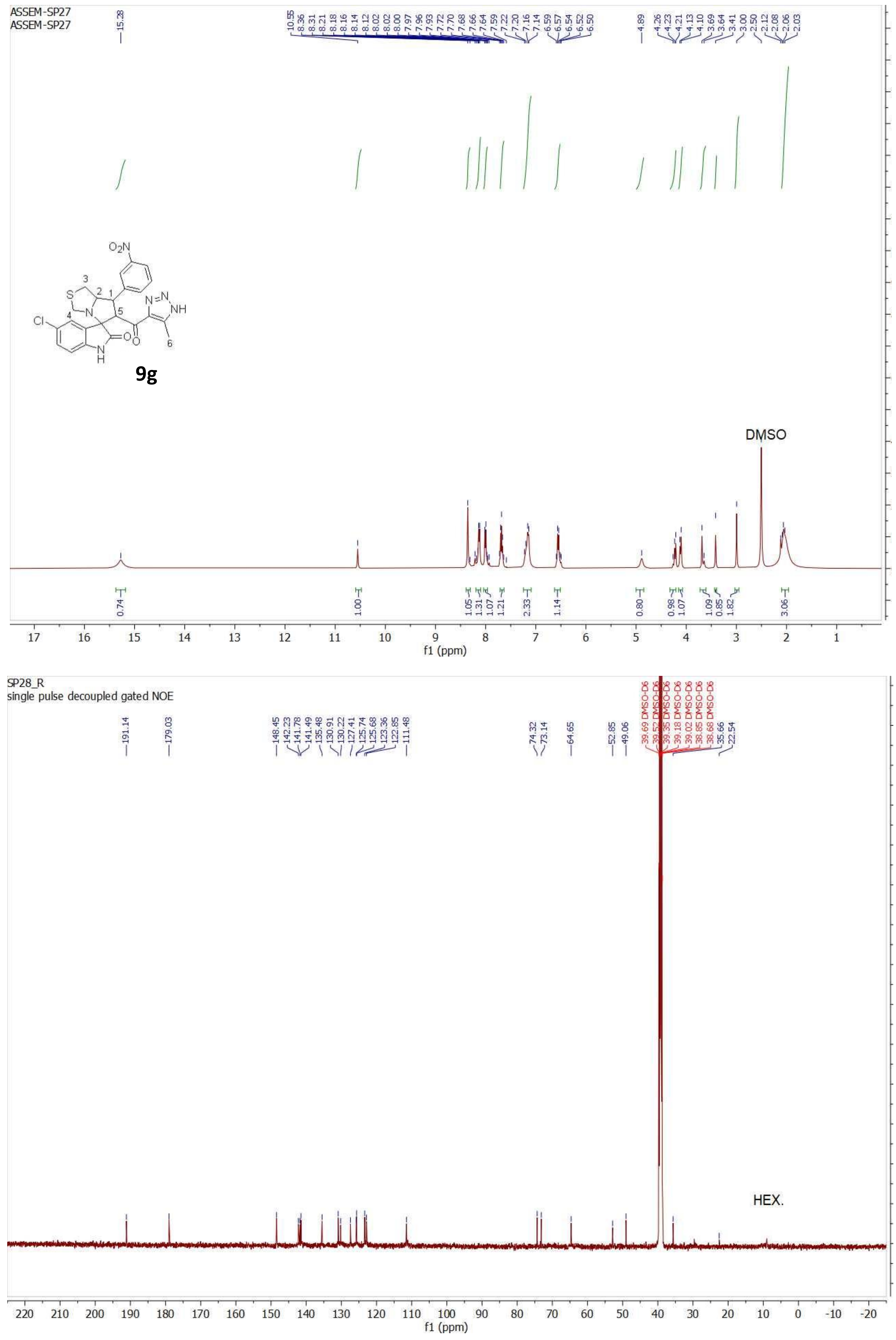

Figure S13: ^{1}H-NMR and ^{13}C-NMR of **9g**

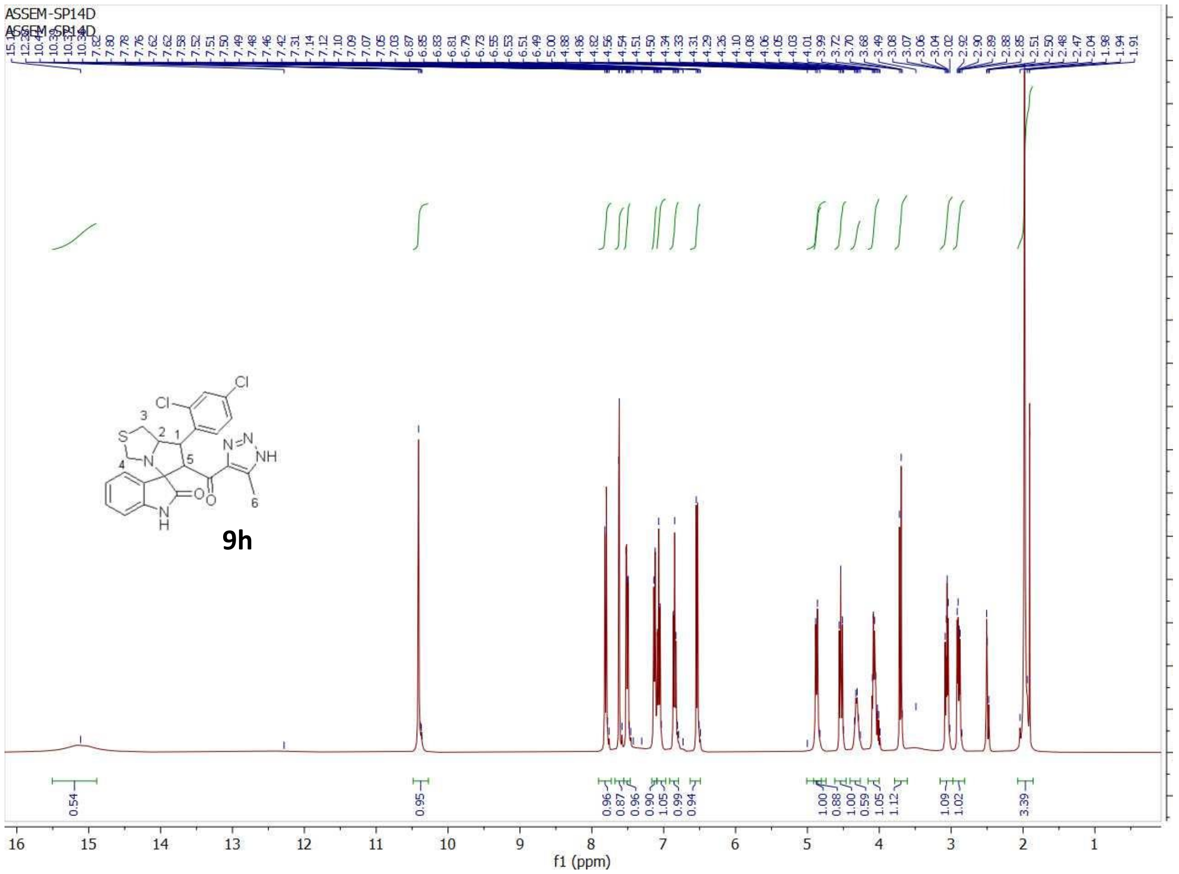

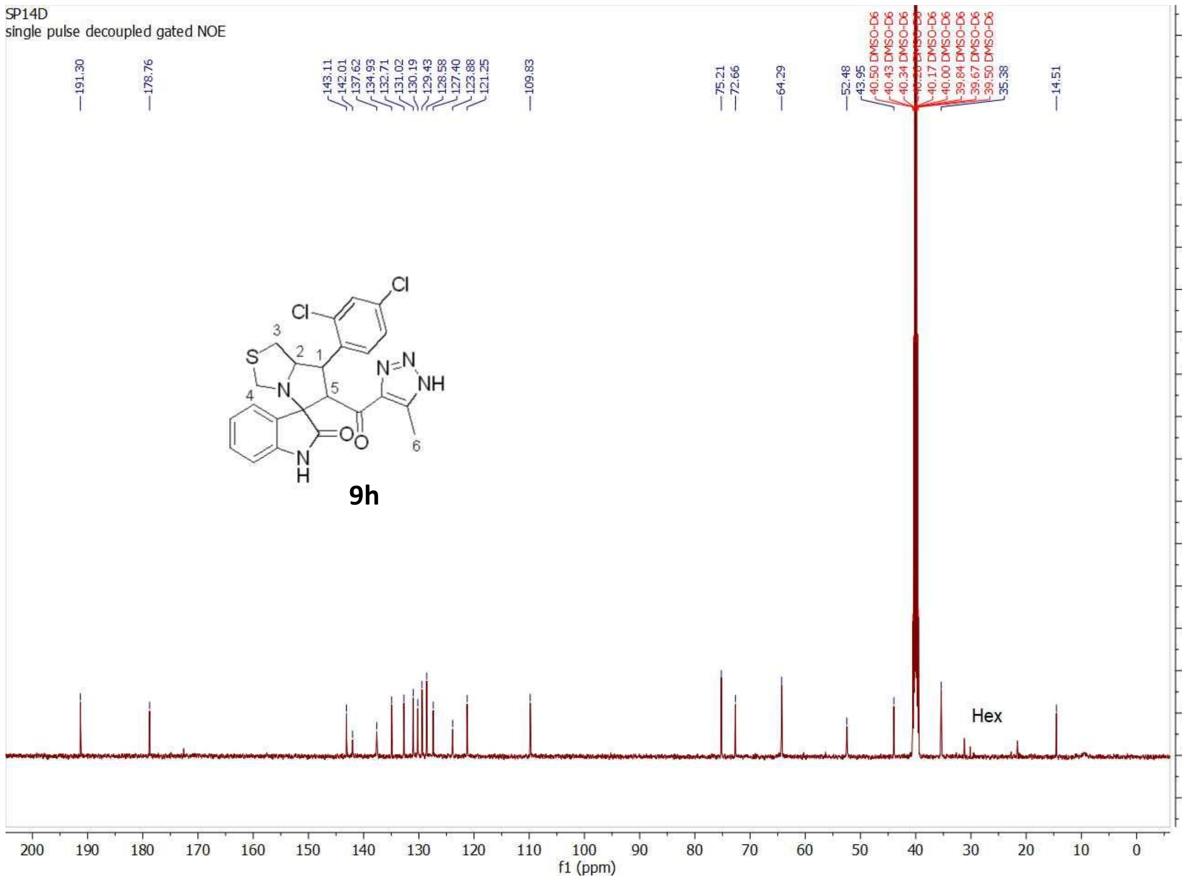

Figure S14: ^{1}H-NMR and ^{13}C-NMR of **9h**

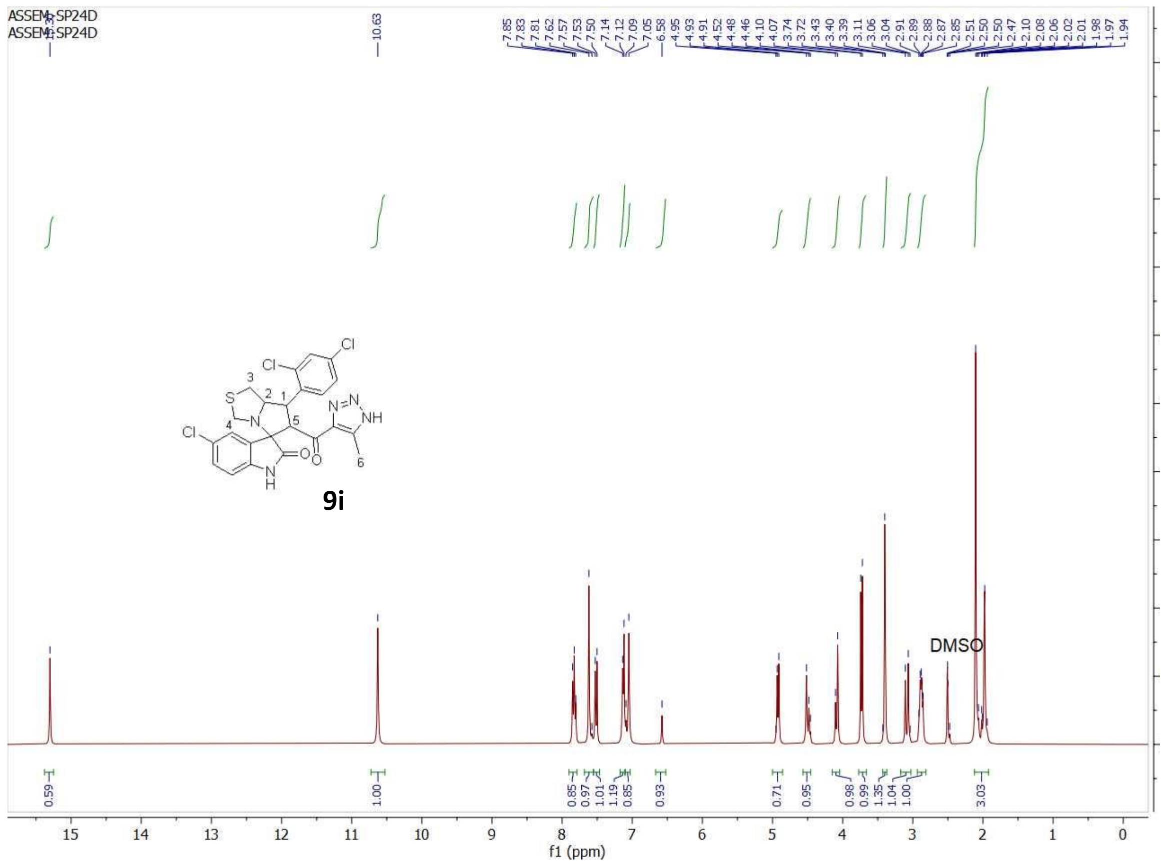

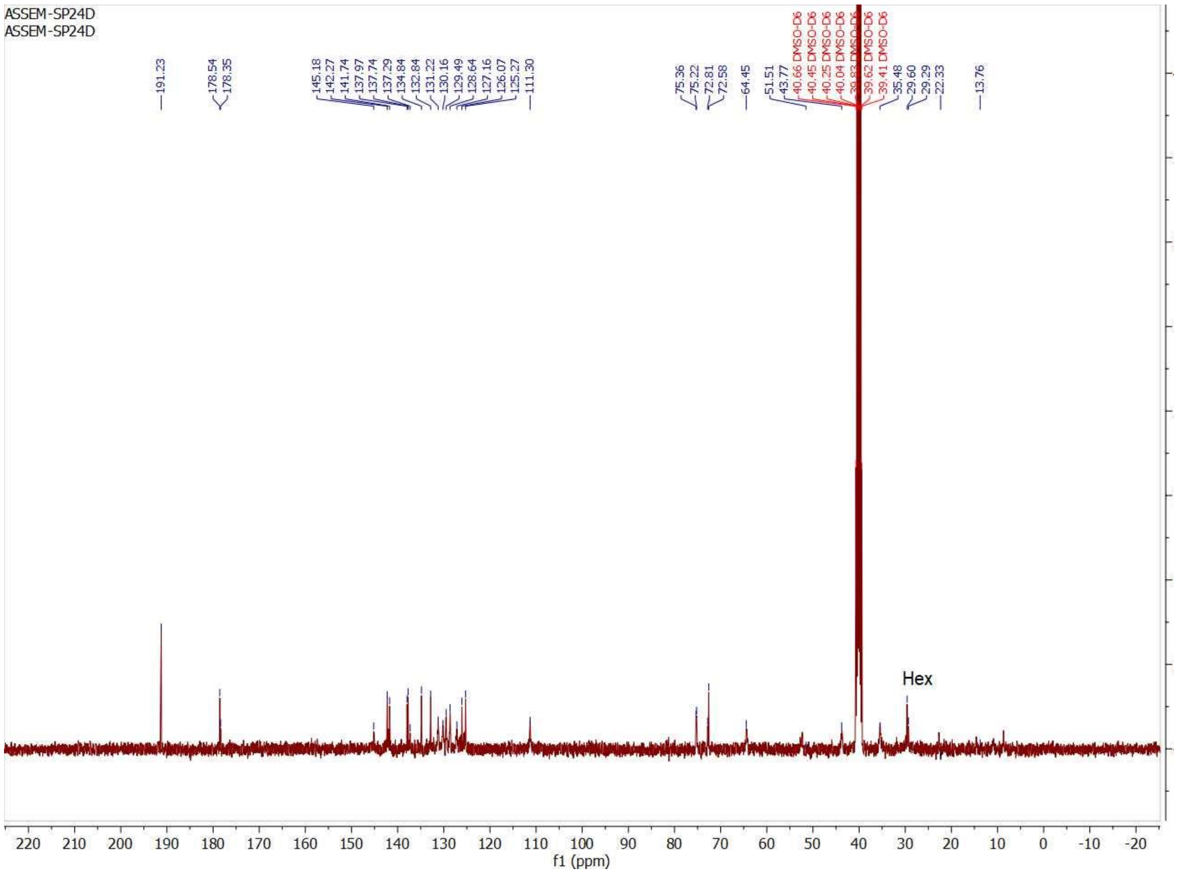

Figure S15: ^{1}H-NMR and ^{13}C-NMR of **9i**
